# The role of cholesterol metabolism and various steroid abnormalities in autism spectrum disorders: A hypothesis paper

**DOI:** 10.1002/aur.1777

**Published:** 2017-04-12

**Authors:** Christopher Gillberg, Elisabeth Fernell, Eva Kočovská, Helen Minnis, Thomas Bourgeron, Lucy Thompson, Clare S. Allely

**Affiliations:** ^1^ Gillberg Neuropsychiatry Centre, Sahlgrenska Academy, University of GothenburgGothenburgSweden; ^2^ Barts and London School of Medicine, Queen Mary University of London, Blizard Institute58 Turner StreetE1 2ABLondon; ^3^ Institute of Health and Wellbeing, University of Glasgow, RHSC YorkhillGlasgowScotlandG3 8SJUnited Kingdom; ^4^ Institut Pasteur, Human Genetics and Cognitive Functions UnitParisFrance; ^5^ CNRS UMR 3571: Genes, Synapses and Cognition, Institut PasteurParisFrance; ^6^ Université Paris Diderot, Sorbonne Paris CitéHuman Genetics and Cognitive FunctionsParisFrance; ^7^ FondaMental FoundationCréteilFrance; ^8^ School of Health SciencesUniversity of SalfordManchesterEngland; ^9^ Honorary Research Fellow in the College of MedicalVeterinary and Life Sciences affiliated to the Institute of Health and Wellbeing at the University of Glasgow

**Keywords:** autism, cholesterol, cortisol, estrogens, steroid hormones, testosterone, vitamin D

## Abstract

Based on evidence from the relevant research literature, we present a hypothesis that there may be a link between cholesterol, vitamin D, and steroid hormones which subsequently impacts on the development of at least some of the “autisms” [Coleman & Gillberg]. Our hypothesis, driven by the peer reviewed literature, posits that there may be links between cholesterol metabolism, which we will refer to as “steroid metabolism” and findings of steroid abnormalities of various kinds (cortisol, testosterone, estrogens, progesterone, vitamin D) in autism spectrum disorder (ASD). Further research investigating these potential links is warranted to further our understanding of the biological mechanisms underlying ASD. ***Autism Res*** *2017*. © 2017 The Authors Autism Research published by Wiley Periodicals, Inc. on behalf of International Society for Autism Research. ***Autism Res** 2017, 10: 1022–1044*. © 2017 International Society for Autism Research, Wiley Periodicals, Inc.

## Introduction

Between 10 and 35% of autism spectrum disorder (ASD) cases have a known major risk factor or identified aetiology, meaning that in the majority of individuals with ASD the exact causes remain unknown [Coleman & Gillberg, 2012]. Numerous potential factors including specific genetic, metabolic, infectious, and environmental factors are being explored [Muhle, Trentacoste, & Rapin, 2004]. One intriguing hypothesis is that ASD is a “whole body disorder” [Herbert, 2010], involving metabolic pathways that are expressed across the whole body [Schengrund, Ali‐Rahmani, & Ramer, 2012].

**Figure 1:**
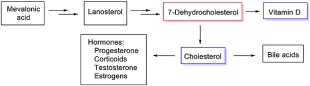
Figure shows the branching point in the metabolism is 7‐dehydrocholesterol (7DHC) from which both VitD and cholesterol are formed. 7DHC, 7‐dehydrocholesterol; VitD, vitamin D.

ASD is diagnosed much more often in boys than in girls and there has long been speculation and controversy about the role of “the male brain” and sex hormones (particularly testosterone) in the pathogenetic chain of events leading to the clinical disorder and to “autistic traits” [Bejerot & Eriksson, 2014; Crider, Thakkar, Ahmed, & Pillai, 2014; Gillberg, 1991; Posserud, Lundervold, & Gillberg, 2006; Wing, 1981]. ASD has also more recently been linked to stress and stress hormone levels [Bitsika, Sharpley, Sweeney, & McFarlane, 2014], and to vitamin D (VitD) insufficiency [Kočovská, Fernell, Billstedt, Minnis, & Gillberg, 2012; Kočovská et al., 2014; Fernell et al., 2015], perhaps suggesting a more general link with steroid hormones rather than one that would be connected specifically with the influence of sex hormones on the developing brain. Finally, a low level of cholesterol—the “precursor” of all the steroid hormones with the exception of VitD—has been indirectly linked to autism in a variety of studies of autism‐associated medical syndromes, ranging from Smith‐Lemli‐Opitz syndrome [Lee & Tierney, 2011] to the Fragile‐X‐syndrome [Berry‐Kravis et al., 2015]. Research to date provides some support for the hypothesis that defective cholesterol homeostasis may be responsible for the onset of ASD in some children, suggesting that it is worth investigating whether cholesterol may be a useful biomarker for certain ASD subtypes [Woods, Wormwood, Wetie, Ryan, & Darie, 2013]. A good overview of steroidogenic enzymes in the pathway from cholesterol to active steroid hormones can be found in a paper by Payne and Hales [2004]. It is important to highlight that VitD is the only steroid hormone which is not metabolically synthesized from cholesterol like all other steroid hormones. The branching point in the metabolism is 7‐dehydrocholesterol (7DHC) from which both VitD and cholesterol are formed (see Figure 1 where the double arrows represent multiple steps within the metabolic process).

Taken together, we believe that these seemingly divergent lines of evidence warrant further scrutiny. Based on evidence from the relevant literature we present a hypothesis that there is a link between 7DHC, cholesterol, VitD, and steroid hormones on the one hand which subsequently impacts on the development of at least some of the “autisms” [Coleman & Gillberg, 2012] on the other.

## Method Section

The present review was too multifaceted, complex, and conceptual to adopt a systematic PRISMA (PRISMA, Preferred reporting items for systematic reviews and meta‐analyses) review format [Liberati et al., 2009; Moher, Liberati, Tetzlaff, & Altman, 2009]. However, we were strictly inclusive in the searches conducted on relevant databases (PsycINFO and Pubmed, etc.) and articles subsequently identified as relevant to the present hypothesis paper. We did not selectively include articles because of favorable results or which supported our conceptual arguments. All articles which were identified as relevant were included regardless of findings. Numerous searches were conducted such as: “cholesterol” AND “autism spectrum disorder*” OR ASD OR asperger* OR “autism spectrum condition*” OR autism OR autistic. Searches were also conducted on google scholar using the same search terms used in the database searches.

## Testosterone in Autism Spectrum Disorders

Testosterone is a steroid hormone which is directly metabolized from cholesterol [Durdiakova et al., 2011]. Several studies have investigated the association between testosterone and ASD behavioral symptoms [Krajmer, Jánošíková, Špajdel, & Ostatníková, 2010]. These studies have usually been linked to the extreme male brain theory of ASD [Baron‐Cohen, 2002] which characterizes ASD as an extreme variant of male intelligence [Asperger, 1991; Baron‐Cohen, 2002, 2005]. Numerous studies to date indicate that elevated testosterone levels may be a risk or associated factor in ASD [Auyeung & Baron‐Cohen, 2013]. However, it is still unclear whether ASD is generally associated with high levels of testosterone or if the association is seen only in a small subset of individuals with ASD. Table 1 shows the studies found which have investigated testosterone in ASD.

**Table 1:** Studies Which have Investigated Levels of Testosterone in Autism Spectrum Disorder

Testosterone in autism spectrum disorders			
**Authors**	Sample	Study aims	Main findings
Auyeung et al. [2009]	The Childhood Autism Spectrum Test (CAST) and the Child Autism Spectrum Quotient (AQ‐Child) were used to assess ASD traits and were completed by the women about their children (*n* = 235), ages 6–10 years. Intelligence Quotient (IQ) was measured in a subset of children (*n* = 74).	To examine the relationship between ASD traits and FT levels in amniotic fluid which was measured during routine amniocentesis.	There was a positive association between levels of FT and higher scores on both the CAST and AQ‐Child, independent of sex which supports the hypothesis that prenatal androgen exposure is associated a greater number of ASD traits in children.
Auyeung et al. [2010]	129 typically developing toddlers (age range = 18 and 24 months, mean age = 19.25 months, SD = 1.52 months).	To investigate whether fetal testosterone (FT) is positively correlated with traits of ASD in toddlers aged between 18 and 24 months. FT levels were measured in the amniotic fluid and compared with autistic traits which were assessed using the Quantitative Checklist for Autism in Toddlers (Q‐CHAT).	Study found sex differences in Q‐CHAT scores, with significantly higher scores found in the boys (suggesting a greater number of autistic traits) compared to girls. Moreover, there was a significant positive relationship between levels of FT and autistic traits
Baron‐Cohen et al. [2015]	Control group (*n* = 217) and autism group (*n* = 128). Participants were selected from a population of singleton births between 1993 and 1999 (*n* = 19,677).	To investigate the prediction that fetal steroidogenic activity is elevated in ASD. Specifically, the study measured concentration levels of Δ4 sex steroids (progesterone, 17α‐hydroxy‐progesterone, androstenedione, and testosterone) and cortisol with liquid chromatography tandem mass spectrometry.	Study found that the autism group had elevated levels of all hormones on this latent generalized steroidogenic factor (Cohen's *d* = 0.37, *P* = 0.0009) and this elevation was found to be uniform across ICD‐10 diagnostic label.
Ingudomnukul et al. [2007]	A number of groups including: women with ASD (*n* =54), mothers of children with ASD (*n* = 74) and mothers of typically developing children (*n* = 183)	To examine the rate of testosterone‐related medical conditions in women with ASD and mothers of children with ASD, as part of the “broader autism phenotype.”	Elevated levels of fetal testosterone (FT) show a positive correlation with a variety of ASD traits and an inverse correlation with social development and empathy. Significantly more women with ASD, when compared to controls, were found to have conditions associated with elevated levels of androgen including: hirsutism; bisexuality or asexuality; irregular menstrual cycle; dysmenorrhea; polycystic ovary syndrome; severe acne; epilepsy; “tomboyism” and a family history of ovarian, uterine, and prostate cancers, tumours, or growths. Additionally, compared to mothers of typically developing children, there were significantly more reports of severe acne, breast and uterine cancers, tumors, or growths, and family history of ovarian and uterine cancers, tumours, or growths in the mothers of children with ASD.
Knickmeyer, Baron‐Cohen, Fane et al. [2006]	Sixty individuals with congenital adrenal hyperplasia (CAH) (34 female, 26 male) and 49 unaffected relatives (24 female, 25 male)	To investigate the hypothesis that autistic traits are increased following prenatal exposure to abnormally high levels of testosterone caused by CAH.	Study found a greater number of ASD traits in girls with abnormally high fetal testosterone (FT) levels as a result of CAH compared to their unaffected sisters. These findings have led to the androgen theory of ASD which proposes that elevated levels of FT are a contributory factor in ASD occurrence.
Kosidou et al. [2016]	23,748 ASD cases and 208,796 controls. Children aged 4–17 who were born in Sweden from 1984 to 2007.	A population‐based study to investigate the relationship between maternal diagnosis of polycystic ovary syndrome (PCOS) and the risk of ASD in the offspring.	Study found that maternal PCOS increased the odds of ASD in the offspring by 59%, following adjustment for confounding factors (odds ratio [OR] 1.59, 95% confidence interval [CI] 1.34–1.88). Findings also revealed that the odds of offspring ASD were even greater in mothers with both PCOS and obesity, a condition common to PCOS that is related to more severe hyperandrogenemia (OR 2.13, 95% CI 1.46–3.10). There were no differences in risk estimates between sexes.
Takagishi et al. [2010]	92 healthy, Japanese adults (45 males and 47 females, mean age = 47.9, SD = 12.4; age range = 21–68).	To investigate whether the relationship between testosterone levels and ASD traits found in numerous studies on young children is also supported by data on adults. Indeed, one study examined the relationship between salivary testosterone levels and ASD traits [using the Japanese version of Autism Spectrum Quotient (AQ)] in 92 healthy, Japanese adults.	When males and females were combined into one group, there was a positive correlation between T and AQ. However, this correlation did not occur when correlation analysis by sex was performed. No sex difference in the score of the subscale of attention switching were found in relation to T.
Whitehouse et al. [2012]	184 males (*M* = 20.10 years; SD= 0.65 years) and 190 females (*M* = 19.92 years; SD = 0.68 years). Also, umbilical cord blood was collected from 707 children	To investigate, for the first time, the prospective relationship between umbilical cord testosterone concentrations and characteristics of autism.	Findings suggest that testosterone concentrations from umbilical cord blood are unrelated to autistic‐like traits in the general population. Findings do not exclude an association between testosterone exposure in early intrauterine life and ASD.

CAST, the childhood autism spectrum test; AQ‐Child, child autism spectrum quotient; IQ, intelligence quotient; ASD, autism spectrum disorders; FT, fetal testosterone; *M*, mean; SD, standard deviation; Q‐CHAT, quantitative checklist for autism in toddlers; CAH, congenital adrenal hyperplasia; PCOS, polycystic ovary syndrome; AQ, autism spectrum quotient; T, testosterone.

The “extreme male brain” theory of ASD [Baron‐Cohen, 2002] has generated hypotheses about the role of elevated fetal sex steroids, such as testosterone, in the development of ASD. However, it does not take into account the contribution of other factors such as sex chromosome effects, or the involvement of other factors such as 7‐dehydrocholesterol, cholesterol, or steroid hormones (including cortisol and VitD) that are proximal or parallel to testosterone in the biosynthesis pathways. Individuals with an extra X chromosome, e.g., boys with Klinefelter syndrome (XXY) and girls with Trisomy X), are at increased risk for autism symptoms [Van Rijn et al., 2014]. Moreover, girls with Turner syndrome (X0), i.e., with haploinsufficiency of X chromosome genes, are at increased risk for a number of neurodevelopmental conditions, generally more often occurring in males than in females; autism and ADHD. Moreover, contrary to the “extreme male brain theory,” Bejerot et al. [2012] posit that rather than being characterized by masculinization in both genders, ASD may instead constitute a gender defiant disorder [Bejerot et al., 2012]. This notion is supported by Strang et al. [2014] who observed an over‐representation of individuals with ASD referred for gender identity disorders (GID).

It is noteworthy that the neuroimaging literature has started to address this topic as well and found some correspondence to, for example, the findings of Bejerot et al. [2012] reviewed above. Such findings have been summarized in a recent review by Lai et al. [2017]. For instance, Lai et al. [2013] adopted whole‐brain voxel‐based morphometry in order to investigate the spatial overlap between normative sex/gender differences in regional brain volume and autism‐related regional brain volume changes in adult males and females. Findings revealed that the overall pattern of autism‐related brain changes (in both gray and white matter) in females significantly resemble neural masculinization. Conversely, the autism‐related brain changes (in both gray and white matter) in males was found to be a weaker but still significant level of resemblance to feminization [Lai et al., 2013].

However, it remains controversial and not well understood as to whether GID and ASD are associated or, alternatively, if the behaviors found in individuals with GID are atypical manifestations of restricted, repetitive, and stereotypical behaviors and interests. Prevalence of GIDs among the general population has been suggested to be nearly identical in males and females [Landén, Wålinder, & Lundström, 1996]. Interestingly, de Vries, Noens, Cohen‐Kettenis, van Berckelaer‐Onnes, and Doreleijers [2010] found that more males than females with ASD experienced GID or GD (gender dysphoria) [de Vries et al., 2010] which is inconsistent with the hypothesis that prenatal androgen exposure may increase male‐typical characteristics in individuals with ASD—the extreme male brain theory [Baron‐Cohen, Knickmeyer, & Belmonte, 2005]. (In May 2013, the DSM‐5 [American Psychiatric Association, 2013] outlined a new diagnostic criteria for Gender Dysphoria (GD) which was formerly Gender Identity Disorder [GID, DSM‐IV‐TR]). According to the extreme male brain theory, females with ASD should be more susceptible to developing GD and it also fails to explain why males with ASD would experience GID [De Vries et al., 2010; Kirkovski, Enticott, & Fitzgerald, 2013]. Other factors, including environmental ones (e.g., certain pesticides exposure, long term exposure to toxic chemicals) may increase risk for ASD, which highlights the need for further research using multiple perspectives [Wormwood, Dupree, Darie, & Woods, 2014]. Lastly, research needs to explore the possibility that low levels of cholesterol could be linked to high testosterone. Low levels of cholesterol would definitely be linked to low levels of estrogens and cortisol.

There have been two recent studies which have shown the risk roles of prenatal steroids (including androgens) for childhood ASD. Baron‐Cohen et al. [2015], used the Danish Historic Birth Cohort and Danish Psychiatric Central Register and identified all amniotic fluid samples of males who were born between the years 1993–1999 and who also had later received ICD‐10 diagnoses of autism, Asperger Syndrome, or PDD‐NOS (*n* = 128). This group was compared to a matched typically developing control group. Baron‐Cohen et al. [2015] tested the concentration levels of Δ4 pathway sex steroids (progesterone, 17α‐hydroxy‐progesterone, androstenedione, and testosterone) and cortisol. The findings revealed that there were elevated levels across all hormones in the ASD group. Interestingly, this elevation of hormones was uniform across ICD‐10 diagnostic subgroups. This study is the first direct support for the existence of elevated fetal steroidogenic activity in ASD [Baron‐Cohen et al., 2015]. In their other study, Kosidou et al. [2016] investigated whether maternal polycystic ovary syndrome (PCOS), a condition associated with excess androgens, would increase the risk of the development of ASD in the children of mothers with this syndrome. They carried out a matched case‐control study nested within the total population of Sweden (children aged 4–17 who were born in Sweden between the years of 1984 and 2007). The study comprised of 23,748 ASD cases and 208,796 controls. The controls were matched by birth month and year, sex, and region of birth. Findings from the study revealed that maternal PCOS increased the odds of ASD in the offspring by 59%. The odds of offspring ASD were further increased among mothers with both PCOS and obesity, a condition common to PCOS that is related to more severe hyperandrogenemia. There were no significant differences between males and females regarding the risk associated with PCOS. In sum, maternal diagnosis of PCOS increased the risk of the offspring of these mothers developing ASD [Kosidou et al., 2016]. This is consistent with the findings from the same group of researchers that maternal PCOS can result in an increased risk for neurodevelopmental disorders such as attention‐deficit/hyperactivity disorder (ADHD) [Kosidou et al., 2016].

Given that the “extreme male brain” theory of ASD [Baron‐Cohen, 2002] has investigated the role of elevated fetal sex steroids, such as testosterone, in the development of ASD, it would be of clinical importance to take this investigation even further and explore the possible contribution of other factors such as sex chromosome effects, or the involvement of other factors such as cholesterol or steroid hormones (including cortisol and VitD) that are proximal or parallel to testosterone in the biosynthesis pathways. These potential factors will be explored in the next sections of this hypothesis paper.

## Estrogens in Autism Spectrum Disorder

One posited explanation for the “extreme male brain theory” is that there may have been an insufficient supply of estrogens from the mother during embryonic development, coupled with the fact that females are exposed in utero and postnatally to higher levels of estrogen and also to oxytocin, and to lower levels of androgens [Pfaff, Rapin, & Goldman, 2011]. This theory of estrogens deficiency as a feature of ASD is consistent with the clinical observation that ASD is associated with reduced cortical thickness in bones [Hediger et al., 2008]. However, this theory is inconsistent with the endocrinological processes which take place. Specifically, it is important to separate the activating effects of testosterone (i.e., the effects exerted by the circulating hormone in adults), which are “acute,” and early organizational effects which are exerted during development, and which are permanent (i.e., remaining also in the absence of the hormone). The former are exerted partly by androgen receptors and may well be influenced by the testosterone versus estrogens ratio (though some of the testosterone effects are in fact mediated by estrogen receptors after the conversion of testosterone to estradiol). The latter, on the other hand, are more or less solely exerted by testosterone acting via estrogen receptors (in males but not in females since testosterone but not estrogens may enter the relevant cells) [Angelopoulou, Lavranos, & Manolakou, 2006]. Given these effects, estrogens may not exert much of an opposing influence. Rather, there have been reports suggesting that administration of exogenous estrogens to pregnant women, leading to estrogen excess, may in fact cause a masculinization of the fetus (though this has also been questioned) [Ehrhardt et al., 1985]. Strifert [2014] has recently hypothesized that estrogens and progesterone, which are used in oral contraceptives, modify the condition of the oocyte producing a potent risk factor for ASD [Strifert, 2014]. See Table 2 for studies which have investigated estrogen levels in ASD.

**Table 2:** Studies Which have Investigated Levels of Estrogen in Autism Spectrum Disorder

Estrogen and autism spectrum disorder			
**Authors**	Sample	Study aims	Main findings
Chakrabarti et al. [2009]	Individuals (*n* = 349). 143 males and 206 females, mean age = 22.5 years, SD = 2.6 years).	To investigate whether genes related to sex steroids, neural growth, and social–emotional behavior are associated with autistic traits, empathy, and Asperger syndrome.	This study found a significant association of the ERβ gene with scores on the Autism Spectrum Quotient and the Empathy Quotient in individuals with ASD.
Crider et al. [2014]	Samples of postmortem middle frontal gyrus tissues from 13 individuals who had received a diagnosis of ASD and 13 controls.	To investigate estrogen receptor beta (ERβ), aromatase (CYP19A1), and ER co‐activators in the middle frontal gyrus in individuals with ASD compared to individuals without ASD.	This study is the first to provide evidence of the dysregulation of ERβ and co‐factors in the brain of individuals with ASD. Molecular alterations within the ER signalling pathway may be one factor underlying the sex difference in ASD. Gene expression was ascertained by qRT‐PCR. A 35% decrease in ERβ mRNA expression in the middle frontal gyrus of ASD subjects was found based on the gene expression analysis. Furthermore, a reduction of 38% in aromatase (CYP19A1) mRNA expression was found in the samples derived from the ASD participants.

SD, standard deviation; *M*, mean; ASD, autism spectrum disorders; ERβ, estrogen receptor beta; CYP19A1, aromatase.

## Cortisol in Autism Spectrum Disorders

The primary stress hormone in humans is cortisol which is released from the adrenal cortices after limbic‐hypothalamic‐pituitary‐adrenocortical (HPA) axis activation in response to physiological and/or psychological stress [Hennessey & Levine, 1979; Herman & Cullinan, 1997]. Cortisol exhibits a normal circadian rhythm, increasing sharply and peaking in early morning (the cortisol awakening response [CAR]) typically around 30 min after waking and the levels of cortisol then decline throughout the remainder of the day. In addition, cortisol can be activated by threat which is real or simply perceived by the individual [Corbett & Simon, 2013]. It has been understood for some time that individuals with ASD are impaired in their ability to adapt to novel and rapidly changing situations [Kanner, 1943]. This has prompted research to study the stability of the HPA axis which has highlighted heightened reactivity in response to different stressors [Corbett, Mendoza, Abdullah, Wegelin, & Levine, 2006; Corbett, Schupp, & Lanni, 2012; Richdale & Prior, 1992; Tordjman et al., 1997]. Cortisol therefore has a role in both circadian rhythms (e.g., sleep) and a stress responsivity, both of which can be impaired in ASD.

Studies to date investigating the diurnal regulation of cortisol in individuals with ASD compared to typically developing controls [e.g., Corbett et al., 2006; Corbett, Mendoza, Wegelin, Carmean, & Levine, 2008; Richdale & Prior, 1992] have produced inconsistent findings. The lack of consistent findings across the studies investigating the CAR in individuals with ASD compared to those without a diagnosis of ASD [e.g., Brosnan, Turner‐Cobb, Munro‐Naan, & Jessop, 2009 vs. Zinke, Fries, Kliegel, Kirschbaum, & Dettenborn, 2010] may be attributable to differences across many factors including: age/stage of developmental (adolescence vs. childhood), the diagnostic criteria used, and diagnostic distinctions (e.g., Asperger syndrome vs. High Functioning Autism [HFA]), the setting from which the sample was derived (institutional living vs. home) or medication status of the individuals (off vs. on medication) [Corbett & Schupp, 2014]. See Table 3 for studies which have investigated cortisol in ASD.

**Table 3:** Studies Which have Investigated Cortisol in Autism Spectrum Disorder

Cortisol in autism spectrum disorders			
Authors	Sample	Study aims	Main findings
Brosnan et al. [2009]	All male and aged between 11 and 16 years and medication free	To investigate cortisol awakening response (CAR) magnitude over 2 days in 20 adolescent males with Asperger Syndrome (AS) from an institutional setting compared to 18 typically developing (TD) youth from the community.	While a significant CAR was evidenced in the TD control group (28%), this was not the case for those with AS (where CAR was only seen in 5%). In both groups, there was evidence of a normal diurnal decrease in cortisol.
Corbett et al. [2008]	Circadian rhythms of cortisol were estimated in 22 children with and 22 children without autism via analysis of salivary samples collected in the morning, afternoon, and evening over 6 separate days. The 44 subjects were predominantly male children between 6.5 years and 12 years of age (mean age 9.08 y), 22 of whom were diagnosed with autism (1 female child) according to a strict diagnosis and 22 of whom were neurotypical children (3 female children).	To replicate and extend their previous findings showing variable circadian rhythm and significant elevations in cortisol following exposure to a novel stimulus (mock magnetic resonance imaging [MRI]).	In children with ASD, a flattening of the diurnal slope over time was found in which morning cortisol levels are diminished and evening cortisol levels are elevated.
Corbett et al. [2009]	44 predominantly male children between 6‐years and 12‐years of age (mean age 9.08 years), 22 diagnosed with autism (1 female) and 22 neurotypical children (3 females).	To investigate plausible explanatory factors which may contribute to the variability in limbic hypothalamic pituitary adrenocortical (LHPA) regulation and responsivity in children with autism.	Diminishing morning cortisol was associated with sensory sensitivity and elevated evening cortisol levels were associated with poor adaptation to changes.
Corbett and Schupp [2014]	94 prepubertal male children between eight and 12 years with ASD (*n* = 46) and typical development (TD, *n* = 48)	Over three diurnal cycles, salivary samples were collected involving two morning samples: M1: Immediately upon Waking and M2: 30‐min Post Waking (M2−M1 = cortisol awakening response (CAR).	Findings revealed no significant differences, over the three days of testing, on the CAR between the groups based on magnitude, variability or the sequence. Whether the child or adult criterion was used, there were still no significant differences in the proportion of children exhibiting a CAR across the groups. So in sum, although there are differences in the regulation and responsivity of cortisol between the individuals with ASD and those without, there are no differences in the CAR between the groups.
Lydon et al. [2015]	61 children and adolescents with a diagnosis of ASD (age range from 3 to 18 years).	To investigate the relationship between a parent‐reported measure of stress, a physiological measure of stress (diurnal salivary cortisol) and a variety of topographies of challenging behavior in 61 children and adolescents with a diagnosis of ASD.	The findings indicated that there are comparable levels of stress present among children with ASD and their typically developing peers. However, for a subset of the children with ASD, stereotyped behavior may be an indicator of elevated levels of cortisol.
Tordjman et al. [2014]	55 low‐functioning children and adolescents with ASD (11.3 ± 4.1 years‐old) and 32 typically developing controls (11.7 ± 4.9 years‐old) who were age‐, sex‐, and puberty state‐matched.	To investigate cortisol levels in 55 low‐functioning children and adolescents with ASD (11.3 ± 4.1 years‐old) and 32 typically developing controls (11.7 ± 4.9 years‐old) who were age‐, sex‐, and puberty state‐matched. The Autism Diagnostic Observation Schedule (ADOS) was used to conduct behavioral assessment and salivary samples to measure levels of cortisol were collected over 24 hr.	Significantly higher levels of salivary cortisol were exhibited at all time‐points in the individuals with ASD. Compared to the typically developing control group, individuals with ASD also exhibited greater variances of salivary and urinary cortisol. A significant association was also found between salivary cortisol levels and impairments in social interaction and verbal language. Similar levels of overnight urinary cortisol excretion were found between the two groups which suggests that there are not abnormalities in the functioning of the basal HPA axis in individuals with low‐functioning ASD.
Yang et al. [2015]	ASD (*n* = 35), mean age 10.63 (SD = 2.64) (28 males and 7 females) and controls (*n* = 32), mean age 11.19 (SD = 2.61) (25 males and 7 females).	To investigate diurnal variation of cortisol (cortisol VAR), interleukin‐6 (IL‐6) and tumor necrosis factor‐alpha (TNF‐a) in order to determine whether they may be clinically useful biomarkers for ASD.	Findings revealed that, compared to the healthy controls, individuals with ASD exhibited a lower level of cortisol VAR, higher level of IL‐6 and TNF‐a. There was a significant correlation between levels of cortisol VAR, IL‐6, and TNF‐a and severity of ASD symptoms as measured using CARS. Analysis showed that cortisol VAR, IL‐6, and TNF‐a are possible biomarkers for ASD and that when cortisol VAR, IL‐6, and TNF‐a are combined, they demonstrate the highest sensitivity and specificity for ASD.
Zinke et al. [2010]	15 children with high‐functioning autism (HFA) and 25 TD children (all aged between 6 and 12 years of age). Seven of the children with HFA had comorbid conditions and six were on medication.	To investigate the CAR over two days in 15 children with high‐functioning autism (HFA) and 25 TD children	Frequency of the CAR was similar between children with ASD and those without (80 vs. 88%, respectively). No significant differences based on the adult criterion [Wust et al., 2000] were exhibited in children with ASD compared to those without a diagnosis of ASD (73 vs. 84%, respectively).

SD, standard deviation; M, mean; CAR, cortisol awakening response; AS, Asperger's syndrome; TD, typically developing; VD, vitamin D; MRI, magnetic resonance imaging; ASD, autism spectrum disorders; LHPA, limbic hypothalamic pituitary adrenocortical; Cortisol VAR, variation of cortisol; IL‐6, interleukin‐6; TNF‐a, tumor necrosis factor‐alpha; HFA, high‐functioning autism.

## Vitamin D in Autism Spectrum Disorders

"Vitamin D” (VitD) is in fact a hormone rather than a vitamin, comprising a group of several fat‐soluble steroid molecules which are transformed through metabolic cascade into its active form (e.g., the multifunctional hormone Calcitriol). VitD3 (cholecalciferol) is primarily involved in calcium physiology and bone metabolism [Christodoulou, Goula, Ververidis, & Drosos, 2013], but also in modulating immune function, cell proliferation and apoptosis, brain development and function, and has also been found to have neuroprotective properties [Holick, 2007].

Sunlight exposure is known to impact on VitD synthesis and has also been explored in relation to ASD. The notion of a potential association between VitD and ASD was derived from a number of observational studies conducted in Northern Europe and North America which all observed higher rates of ASD in dark‐skinned children [Barnevik‐Olsson, Gillberg, & Fernell 2008; Cannell, 2008; Eyles, 2010; Gillberg, Schaumann, & Gillberg, 1995; Goodman & Richards, 1995; Keen, Reid, & Arnone, 2010]. In these Northern areas, there is less intensive solar radiation throughout the year. Individuals who are dark‐skinned require greater levels of UV ray exposure in order to synthesize VitD [Clemens, Adams, Henderson, & Holick, 1982], leading to an increased risk for VitD deficiency. A higher prevalence of ASD at higher latitudes was also found [Grant & Soles, 2009], and the rate of ASD in these higher latitudes was even higher again in dark‐skinned offspring born to immigrant mothers, particularly the mothers who came from East Africa to Northern Europe [Dealberto, 2011]. More recently, an ecological study carried out by Grant and Cannell [2013] in the United States investigated the association between sun exposure and rates of ASD. A negative correlation between ASD prevalence and solar UV‐B doses**—**measured using Total Ozone Mapping Spectrometer [Leffell & Brash, 1996]—was found. Cannell and Grant have also suggested that insufficient VitD is a factor in inducing ASD and Cannell has advocated the use of oral VitD supplementation to reduce the risk of ASD [Cannell, 2008, 2013; Cannell & Grant, 2012; Grant & Cannell, 2013]. VitD supplementation should be given both in pregnancy and possibly also during the child's early life.

One recent study highlighted a link between VitD and serotonin production [Patrick & Ames, 2014]. It was found that the VitD hormone activates the gene which produces the enzyme tryptophan hydroxylase 2 (TPH2) that converts the essential amino acid tryptophan to serotonin in the brain. This indicates that adequate levels of VitD may be needed to produce serotonin in the brain where it shapes the structure and wiring of the brain, acts as a neurotransmitter, and affects social behavior. Evidence was also found that the gene that makes the enzyme tryptophan hydroxylase 1 (TPH1) is inhibited by VitD hormone, which subsequently stops the production of serotonin in the gut and other tissues, where, when found in excess, it promotes inflammation [Patrick & Ames, 2014].

In summary, VitD deficiency does appear to exist in at least a subgroup of individuals with ASD but whether the VitD deficiency in itself contributes to ASD is unclear. A review of the literature on VitD deficiency and ASD emphasized the need for the recognition of and for further research into the potential crucial involvement of VitD in ASD [Kočovská et al., 2012]. See Table 4 for studies which have investigated levels of VitD in ASD. VitD is the only steroid hormone that is not metabolically synthesized directly from cholesterol, but from its “predecessor” 7‐hydrocholesterol (7DHC). We have found no empirical evidence of high testosterone levels in low vitamin D production from 7DHC. However, if 7DHC is not metabolized into VitD (such as in the absence of sunlight exposure or if the enzymatic activity of the final steps of the calcitriol synthesis is impaired, or if its degradation is enhanced), then more cholesterol would be available for production of higher levels of testosterone and other steroid hormones. Also, low cholesterol levels could result from high turnover of 7DHC to VitD, from primarily low levels of mevalonic acid or from the impaired enzymatic activity mentioned above.

**Table 4:** Studies Investigating Levels of Vitamin D in Autism Spectrum Disorder

Vitamin D in autism spectrum disorders			
Authors	Sample	Study aims	Main findings
Azzam et al. [2015]	A prospective case–control 6‐month study was carried out including 21 children with ASD who were randomly assigned to one of two groups.	The patients in group I were administered a daily oral dose of vitamin D3 and the patients in group II were not administered any supplements. Symptoms of ASD were measured both presupplement and postsupplement using the Childhood Autism Rating Scale, social IQ, and Autism Treatment Evaluation Checklist.	Both groups exhibited improved ASD symptoms but the improvement found in the supplemented group did not differ significantly from that of the nonsupplemented group. Azzam et al. [2015] have administered 2,000 IU per day for 6 months (*n* = 21) and reported no changes on CARS scores. This treatment resulted only in a moderate improvement in children with ASD from their presupplementation average levels of 47 nmol/L to their post supplementation average levels of 70 nmol/L.
Bener, Khattab, and Al‐Dabbagh [2014]	254 children with ASD (mean age 5.51, SD = 1.58) and 254 healthy control children (mean age, 5.76 SD = 1.56)	To investigate the association between VitD and ASD, and the difference in level of VitD in children with ASD and children without ASD.	VitD deficiency was greater in children with ASD when compared to healthy children and providing infants with VD supplements may be a more effective intervention for lowering the risk of ASD. Compared to the control children, a lower level (mean value) of VitD in the children with ASD was found. Of total 254 children with ASD, 14.2% had severe VitD deficiency, 43.7% were moderately insufficient, 28.3% were mildly insufficient, and sufficient levels of VD was found in only 13.8% of the children with ASD. Of the 254 of control children 8.3% were found to have a severe VitD deficiency, 37% were moderately insufficient, 37.4% were mildly insufficient, and sufficient levels were found in only 17.3%. Serum level of VitD was also significantly different between the children with ASD and control children.
De Souza‐Tostes, Polonini, Gattaz, Raposo, and Baptista [2012]	24 children (18 male and 6 female, mean age = 7.4 ± 2.7 years) diagnosed with ASD.	To confirm previous evidence suggesting an association between autism and low VitD serum levels.	Findings showed that the serum levels of 25‐OHD were lower in children with autism compared to typically developing participants.
Fernell et al. [2015]	47 Gothenburg sibling pairs with mixed ethnicities and 11 Stockholm sibling pairs with Somali background.	The aim of the present study was to address the emerging hypothesis that low levels of VitD at birth increase the risk for ASD. First study whose findings excluded ASD‐related lifestyle mechanisms as a possible theory for deficient 25(OH)D levels given that samples were taken shortly after birth.	Collapsed group of children with ASD had significantly lower VitD levels compared with their siblings. VitD deficiency was found in both the children with ASD with African/Middle East background and their non‐ASD siblings. These findings are consistent with the hypothesis that during pregnancy, developmental VD deficiency may be one of the factors involved in increasing the risk of ASD in the child.
Gong et al. [2014]	48 confirmed ASD cases and 48 age‐matched and sex‐matched controls. Mean age (%) for ASD group = 40 (83.3) Mean age (%) for control group = 40 (83.3)	To investigate the serum 25(OH) D levels in Chinese children with ASD.	The mean serum 25(OH) D levels were significantly lower in children with ASD compared to the children without ASD. There was a significant negative relationship between circulating serum 25(OH) D levels and the severity of autism evaluated according to Childhood Autism Rating Scale scores, after adjustment for the possible covariates. Lower 25(OH) D levels may be independently associated with severity of ASD among Chinese patients, and lower serum 25(OH) D levels could be considered as an independent risk factor for ASD.
Humble et al. [2010]	Mean age 36.5 of group of individuals with ASD (*n* = 10). 40% were females.	To investigate 25(OH)VitD levels of individuals with ASD (including other psychiatric conditions).	Patients with ADHD had unexpectedly low intact parathyroid hormone (iPTH) levels. Middle East, South‐East Asian, or African ethnic origin, being a young male and having a diagnosis of ASD or schizophrenia predicted low 25‐OHD levels.
Jia et al. [2015]	Case study of a 32‐month‐old boy with ASD and vitamin D3 deficiency.	Investigating Vitamin D/D3 supplementation in a case of deficiency.	Following vitamin D3 supplementation, the boy's core ASD symptoms were significantly reduced indicating that vitamin D3 may be one of the factors contributing to the etiology of ASD.
Kočovská et al. [2014]	The case group consisting of a total population cohort of 40 individuals with ASD (aged 15–24 years) were compared to 62 typically‐developing siblings and their 77 parents and also 40 healthy age and gender matched comparisons.	To investigate, using a cross‐sectional population‐based study conducted in the Faroe Islands, levels of 25‐Hydroxyvitamin D3 (25(OH)D3) in a case group of a total population cohort of 40 individuals with ASD.	The ASD group were found to have a significantly lower 25(OH)D3 when compared to the 25(OH)D3 level in their 62 typically developing siblings and their 77 parents, as well as being significantly lower than 40 healthy age and gender matched comparison cases. A trend also revealed that males had lower 25(OH)D3 levels compared to females. Interestingly, there was no association between 25(OH)D3 and age, month/season of birth, IQ, various subcategories of ASD and, most interestingly, Autism Diagnostic Observation Schedule scores.
Meguid et al. [2010]	The mean age ± standard deviation (SD) of the children with ASD was 5.3 ± 2.8 years. Controls included 42 age‐matched randomly selected healthy children of the same socioeconomic status (mean age ± SD, 6.1 ± 1.8 years).	To measure the potential role of VitD in ASD through serum level assessment.	Findings showed that the children with ASD exhibited a significantly lower 25(OH)D and 1,25(OH)2D compared to controls. The children with ASD also exhibited significantly lower calcium serum values compared to the controls. A significant positive correlation was found between 25(OH)D and calcium within the children with ASD.
Molloy, Kalkwarf, Manning‐Courtney, Mills, and Hediger [2010]	Three groups of Caucasian males age 4–8 years old: (1) ASD and an unrestricted diet (*n* = 40), (2) ASD and a casein‐free diet (*n* = 9), and (3) unaffected controls (*n* = 40).	To examine the plasma 25(OH)D concentration levels across three groups (1) ASD and an unrestricted diet, (2) ASD and a casein‐free diet, and (3) unaffected controls.	A total of 54 (61%) of the children in the entire cohort had a plasma 25(OH)D concentration of less than 20 ng/mL Children with and without ASD have low plasma concentrations of 25(OH)D. There were no significant group differences. 25(OH)D concentrations do not differ between children with ASD and typically developing controls.
Mostafa and Al‐Ayadhi [2008]	50 children with ASD, aged between 5 and 12 years, and 30 healthy‐matched children.	This is the first study to investigate the relationship between serum levels of 25‐hydroxy VitD and anti‐myelin‐associated glycoprotein (anti‐MAG) auto‐antibodies in children with ASD.	Children with ASD had significantly lower serum levels of 25‐hydroxy VitD than healthy children with 40 and 48% being VitD deficient and insufficient, respectively. Serum 25‐hydroxy VitD had significant negative correlations with Childhood Autism Rating Scale. Increased levels of serum anti‐MAG auto‐antibodies were found in 70% of children with ASD.
Saad et al. [2015]	122 children with ASD (3–9 years of age, mean age = 5.09). 100 control cases (3–9 years of ages, mean age = 4.88).	To investigate individuals with ASD VitD status compared to controls and the relationship between VitD deficiency and the severity of ASD. Also, to conduct an open trial of VitD supplementation in children with ASD.	57% of the patients had VitD deficiency, and 30% had VitD insufficiency. Mean 25‐OHD levels in patients with severe ASD were significantly lower compared to those with mild/moderate ASD. Serum 25‐OHD levels had significant negative correlations with Childhood Autism Rating Scale (CARS) scores. Of the ASD group, 106 patients with low‐serum 25‐OHD levels (<30 ng/mL) participated in the open label trial. They received VitD3 (300 IU/kg/day not to exceed 5,000 IU/day) for 3 months. 83 participants completed 3 months of daily VitD treatment. Collectively, 80.72% (67/83) of participants who received VitD3 treatment had significantly improved outcome, mainly in the sections of the CARS and aberrant behavior checklist subscales that measure behavior, stereotypy, eye contact, and attention span.
Schmidt et al. [2015]	ASD (*n* = 474) and typical development (TD, *n* = 281). Age range for both groups = 24–60 months.	To investigate the associations (if any) between ASD and common, functional polymorphisms in VitD pathways.	Preliminary evidence that paternal and child VitD metabolism may contribute to some degree in the etiology of ASD. Paternal VDR TaqI homozygous variant genotype was significantly associated with ASD in case–control analysis and there was a trend toward increased risk associated with VDR BsmI. Further analyses identified parental imprinting, with greater effects of paternally derived VDR alleles.
Ucuz et al. [2014]	64 toddlers with developmental delay.	In the initial assessment, a psychiatric examination and developmental tests were carried out and VitD was measured.	Individuals found to have low VitD levels in the initial assessment received supplementary treatment. Six months later, the same measures were repeated which revealed a significant improvement in ASD symptoms and development scores for the group who received VitD supplementation. Study found evidence which supports the importance of measuring VitD levels and supplementing them if they are low in individuals with ASD.
Utur and Gurkan [2014]	54 young children, aged 3–8 years, with ASD and 54 age and gender matched normal controls.	First preliminary evidence that paternal and child vitamin D metabolism could play a role in the etiology of ASD. To investigate the serum levels of VitD, calcium (Ca), phosphorus (P), alkaline phosphatase (ALP), and folate in children with ASD compared to children without ASD.	Paternal homozygous variant genotypes for the TaqI and BsmI polymorphisms on the VDR gene, and CYP27B1 rs4646536 were associated with increased risk for ASD. The CYP2R1 enzyme catalyses the transformation of vitamin D3 to 25(OH)D3, the main circulating VitD metabolite. The CYP2R1 GG genotype associated with higher risk for ASD in this study was also associated with lower circulating 25(OH)D3 concentrations and with type 1 diabetes in Caucasians. Interestingly, their results suggested that the risk associated with the child's CYP2R1 GG genotype could be counteracted by increasing maternal VitD intake. An inherited gene from a father combined with male sex seems to play an important role and mother's VitD deficiency during pregnancy is an additional risk factor.

SD, standard deviation; *M*, mean; ASD, autism spectrum disorders; VitD, vitamin D; nmol/L, nanomoles (nmol) per litre (L); 25‐OHD, a particular form of vitamin D; 25(OH) D, a particular form of Vitamin D; CARS, the childhood autism rating scale; Ca, calcium; P, phosphorus; ALP, alkaline phosphatase.

## The Cholesterol‐Steroid Hormone Pathways and Autism Spectrum Disorder

In the previous section, we reviewed studies which linked some of the steroid hormones, specifically VitD, with cholesterol. We also found studies linking other steroid hormones, namely testosterone; estrogens and cortisol with ASD. Therefore, it is possible that there may exist a cholesterol‐steroid hormone pathway underlying the development of ASD in some individuals. An increasing number of studies are finding alterations in cholesterol metabolism in some individuals with ASDs [Schengrund et al., 2012]. A number of heritable disorders have been found to be associated with ASD including: fragile X syndrome [e.g., Brown et al., 1982], phenylketonuria [e.g., Baieli, Pavone, Meli, Fiumara, & Coleman, 2003; Kumar, 2014; Saad, Hammad, Abdel‐rahman, & Sobhy, 2013], tuberous sclerosis [Asano et al., 2001; Bolton & Griffiths, 1997; Gillberg, Gillberg, & Ahlsén, 1994; Smalley, 1998; Wiznitzer, 2004], and Smith‐Lemli‐Opitz syndrome (SLOS) [Sikora, Pettit‐Kekel, Penfield, Merkens, & Steiner, 2006; Tierney, Nwokoro, & Kelley, 2000].

SLOS is becoming increasingly recognized as a clinically useful model for understanding the genetic mechanisms underlying ASD [Aneja & Tierney, 2008; Diaz‐Stransky & Tierney, 2014]. SLOS is an autosomal recessive disorder of cholesterol metabolism caused by mutations of the 7‐dehydrocholesterol (7DHC) reductase gene (DHCR7) [Tint et al., 1994], found on chromosome 11q12–13 [Wassif et al., 1998]. In individuals with SLOS (prevalence 1/20,000) insufficient levels of cholesterol are produced in the body and 7DHC accumulates [Aneja & Tierney, 2008; Porter, 2008]. The behavioral phenotype of SLOS exhibits features which overlap with those found in individuals with ASD such as impaired language and social abilities as well as stereotyped and repetitive behaviors [Tierney et al., 2001]. Overall, there appears to be good evidence to suggest that ASD is present in individuals with SLOS. There is evidence of SLOS being present in individuals identified as having ASD [Bukelis, Porter, Zimmerman, & Tierney, 2007]. Numerous studies have investigated the presence of cholesterol abnormalities in individuals with ASD. Tierney et al. [2006], in a cohort of 100 individuals (78 males and 22 females) with ASD (specifically those from families who had more than one individual diagnosed with ASD), investigated the incidence of biochemically diagnosed SLOS using blood samples (the typical diagnostic procedure). None of the samples collected had sterol levels consistent with the full diagnosis of SLOS. Total cholesterol levels lower than 100 mg/dL, which is below the 5th centile for children over age 2 years, were found in 19%. Such findings indicate that, as well as SLOS, there may be additional disorders of sterol metabolism or homeostasis which are related to ASD [Tierney et al., 2006].

Tierney et al. [2001] and Sikora et al. [2006] both found that that 50% of the children with SLOS met autism criteria and Sikora et al. [2006] also found that 75% were on the ASD spectrum. Tierney et al. [2001] found that, in 17 patients with SLOS, 9 met criteria for ASD as assessed using the Autism Diagnostic Interview‐Revised [ADI‐R; Lord at al., 1994], a parental interview. In nine of the 17 patients with SLOS who started cholesterol supplementation prior to the age of 5 years, only 22% met the diagnostic criteria for ASD, compared to 88% of those eight who did not start cholesterol supplementation prior to 5 years of age. Within days of taking cholesterol supplementation, which is before there is a measurable change in the plasma cholesterol or 7DHC level, a number of parents of children with SLOS observed changes in their child's behavior, in particular, a decrease in the number of autistic behaviors. All these findings support the hypothesis that the change in behavior occurs following changes in the levels of cholesterol‐derived steroid *products*, as opposed to the notion that it is the level of cholesterol, which is unable to cross the blood‐brain barrier, that is important [Lee & Tierney, 2011]. The imbalance in cholesterol biosynthesis in individuals with SLOS leads to impairment in myelination [e.g., Fierro, Martinez, Harbison, & Hay, 1977; Opitz, Gilbert‐Barness, Ackerman, & Lowichik, 2002], dendrite differentiation [e.g., Jiang et al., 2010], steroid hormone synthesis [e.g., Marcos, Guo, Wilson, Porter, & Shackleton, 2004; Meljon, Watson, Wang, Shackleton, & Griffiths, 2013] as well as impairment to the functioning of neurotransmitter receptors [e.g., Waage‐Baudet et al., 2003; Wassif et al., 2001] and neurotransmitter levels [e.g., Sparks et al., 2014]. It has been hypothesized that impairment to these processes may be linked to the onset of symptoms of ASD [Bukelis, Porter, Zimmerman, & Tierney, 2007; Lecis & Segatto, 2014], a hypothesis strengthened by studies which have indicated that a significant number of children with ASD have abnormally low levels of plasma cholesterol [Tierney et al., 2006].

Further support for SLOS being a useful model for ASD comes from findings which show that cholesterol affects the oxytocin receptor [Gimpl & Fahrenholz, 2001; Gimpl, Reitz, Brauer, & Trossen, 2008; Reversi, Rimoldi, Brambillasca, & Chini, 2006] which, when absent in male mice, leads to marked impairments in social recognition, obesity, and absence of body temperature control [Nishimori et al., 2008]. Cholesterol is required for the high‐affinity state of the oxytocin receptor [Gimpl & Fahrenholz, 2001]. Theoretically, cholesterol supplementation might increase oxytocin receptor sensitivity and hence the positive effects of oxytocin on social interaction [Aneja & Tierney, 2008]. Not only is oxytocin receptor modulated by cholesterol, G‐protein coupled receptors are also modulated by cholesterol levels [for review see Alexander, Mathie, & Peters, 2011]. Synthesis of oxytocin (OT) occurs in neurons within the hypothalamus as part of a protein precursor, which, as it is transported axonally, is both enzymatically cleaved and amidated [Gainer, Altstein, & Whitnall, 1987]. Previous research found that social impairments found in individuals with ASD are related to changes in plasma OT levels [e.g., Modahl et al., 1998]. Given that social impairments are considered to be the primary symptoms of ASD [Fein, Joy, Green, & Waterhouse, 1996], it was posited that this central OT system may be impaired in individuals with ASD [Modahl, Fein, Waterhouse, & Newton, 1992; Waterhouse, Fein, & Modahl, 1996]. Intranasal administration of OT has been suggested by some small clinical studies to produce social cognition improvements in individuals with ASD [Anagnostou et al., 2012; Andari et al., 2010; Domes et al., 2013; Guastella et al., 2010]. Improvements in the symptoms and behaviors of individuals with ASD have been reported by a number of other small clinical studies [Green & Hollander, 2010; Hollander, 2003; Hollander et al., 2007; Tachibana et al., 2013; Theodosis et al., 2006]. Despite some modest improvement in social stimuli attention and social reciprocal communication, there was no significant impact on primary outcome measures in an open label long‐term trial of OT in adolescents with ASD over a 6 month duration [Tachibana et al., 2013]. The studies to date strongly indicate that improving function in ASD is not as easy as simply administering a course of OT in an intervention [Quattrocki & Friston, 2014]. See Table 5 for studies which have investigated levels of OT in ASD. Quattrocki and Friston [2014] argue that a dysfunction in the oxytocin system, early in life, may account for the development of ASD [Quattrocki & Friston, 2014], at least in some cases.

**Table 5:** Studies Investigating Level of Oxytocin in Autism Spectrum Disorder

Oxytocin receptor modulation and autism spectrum disorder			
Authors	Sample	study aims	Main findings
Al‐Ayadhi [2005]	Seventy‐seven children with ASD participated in the study, 71 males (92.2%) and 6 females (7.8%). Seventy‐seven healthy age and sex matched controls	To investigate plasma levels of oxytocin and vasopressin in children with ASD. Also, to correlate plasma levels of those neuropeptides to the degree of ASD and age of the affected child. Another aim was to explore the role of Pitocin induction in the genesis of ASD.	Oxytocin (OT) and vasopressin levels were also found to be lower in 77 boys with ASD from central Saudi Arabia compared to the boys without ASD. No significant correlation between the severity of ASD, or the age of the affected child and plasma levels of oxytocin or vasopressin was found. A greater incidence of Pitocin‐induced labor among the group with ASD compared to the controls.
Andari et al. [2010]	13 patients with HF‐ASD and 13 matched healthy participants.	To investigate the behavioral effects of oxytocin in 13 participants with ASD.	Study found marked decreased levels of oxytocin (OT) in 13 high functioning autism patients with ASD compared to controls.
Crespi and Hurd [2015]	Questionnaire data and DNA samples from Caucasian undergraduate psychology students (175 males and 309 females) at the University of Alberta and Simon Fraser University.	Study aimed to investigate associations of genetically based indicators of serum oxytocin, and serum testosterone, with measures of autism‐spectrum and schizophrenia‐spectrum cognition in healthy individuals.	Low genetic index of testosterone, a high genetic index of oxytocin (OT), and/or a low ratio of testosterone to OT indices were positively correlated with high imagination (as measured by the Autism Quotient) and high positive and total schizotypy (as measured by the Schizotypal Personality Questionnaire). In the Autism Spectrum Quotient, the Imagination subscale measures areas of social imagination which overlap with mentalistic cognitions such as theory of mind, which previous studies have identified as being association with levels of OT.
Green et al. [2001]	28 males (97 ± 20 months; range, 70–139 months), diagnosed with DSM‐IV autistic disorder through observation and semistructured interview. 31 age‐matched nonpsychiatric controls (106 ± 22 months; range, 74–140 months).	To investigate whether there were changes in OT peptide forms in children with ASD compared to children without ASD.	Findings support the role of OT in ASD. Findings revealed that plasma OT is reduced in children with ASD and also has abnormal associations with social abilities. There are numerous pathways for this association such as alterations in either the brain OT axis, the peptide receptors, and/or in peptide synthesis and processing.
Lakatosova et al. [2013]	8 children with ASD.	To investigate the potential correlations between both peripheral levels of oxytocin (OT) and testosterone with ASD symptomology.	Findings revealed a positive correlation between OT levels and the Adaptation to change category of The Childhood Autism Rating Scale™, Second Edition (CARS‐2) and maladapative behavior scores as measured on The Vineland Adaptive Behavior Scales, Second Edition (Vineland‐II). There were no significant correlations between levels of testosterone and behavioral parameters. Elevated levels of OT were associated with more severe scores of adaptive behavior in ASD patients. It appears that, in individuals with ASD, OT recruits a different mechanism to modulate social behavior compared to the general population.
Miller et al. [2013]	75 preadolescent and adolescent girls and boys ages 8–18: 40 with high‐functioning ASD (19 girls, 21 boys) and 35 typically developing children (16 girls, 19 boys).	To investigate: (1) plasma levels of oxytocin (OT) and arginine vasopressin (AVP) in TYP girls and boys and those with ASD (2) to relate these levels of OT and AVP to measures of social, language, repetitive behavior, & internalizing symptoms	Study did not find any differences between the levels of OT and vasopressin in their sample of 75 patients with ASD compared to age‐matched controls in a United States population. Higher OT values were associated with greater anxiety in all girls. Higher OT values were also found to be associated with better pragmatic language in both boys and girls. AVP levels were positively associated with restricted & repetitive behaviors in girls with ASD. AVP levels were negatively (non‐significantly) associated with these behaviors in boys with ASD.
Modahl et al. [1998]	29 children with ASD and 30 age‐matched typically developing children, all prepubertal.	To investigate whether children with ASD have abnormalities in OT compared to controls using radioimmunoassay for levels of OT.	Children with ASD were found to have plasma OT levels which were significantly lower compared to those in the typically developing group. Moreover, in the typically developing group, OT was found to increase with age, an increase which was not exhibited in the individuals with ASD. Higher scores on social and developmental measures were associated with elevated OT for the children without ASD. However, in the children with ASD, lower scores on social and developmental measures were associated with elevated OT. In the children with ASD who were identified as being ‘aloof’ this association was even more marked.
Taurines et al. [2014]	19 children and adolescents with ASD, all male (mean age 10.7, SD = 3.8 years compared to two other groups, 17 healthy male children (mean age 13.6, SD = 2.1 years) and 19 male children with attention deficit hyperactivity disorder (ADHD) (mean age 10.4, SD = 1.9 years).	To investigate the changes in concentration levels of peripheral OT (OT plasma concentrations) using a validated radioimmunoassay.	Findings revealed a correlation between OT plasma concentrations with number of ASD symptom (as measured using the Autism Diagnostic Observation Schedule) in children with ASD.
Weisman et al. [2015]	All singleton live births in Denmark between 2000 and 2009 (*n* = 557,040) with a follow‐up through 2012. In this cohort, 2,110 children were later diagnosed with ASD.	To investigate oxytocin‐augmented labor and risk for ASD.	Findings showed that augmentation of labor with oxytocin (OT) was only modestly associated with an increased risk for ASD in males only. In the group of males exposed to OT augmentation, 560 were later diagnosed with ASD (incidence rate 103.2 per 100,000 person‐years). In the males not exposed to OT augmentation, 1,177 fulfilled the criteria for an ASD (incidence rate 81.4 per 100,000 person‐years).

SD, standard deviation; *M*, mean; ASD, autism spectrum disorders; OT, oxytocin; HF‐ASD, high‐functioning autism spectrum disorder; DNA, deoxyribonucleic acid; DSM‐IV, diagnostic and statistical manual of mental disorders, 4th edition; CARS‐2, the childhood autism rating scale™, second edition; Vineland‐II, the vineland adaptive behavior scales, second edition; AVP, arginine vasopressin.

Studies investigating the relationships between ASD and cholesterol have had mixed findings. For instance, Schengrund et al. [2012] investigated the correlation between ASD symptoms (using the Autism Diagnostic Interview‐Revised [ADI‐R]) and cholesterol in a group of 16 children with an ASD (one female and 15 males) and 20 typically developing control children (12 females and 8 males). All children were aged between 3 and 8 years of age. Although there was no correlation between scores obtained using the ADI‐R (total score and subscales which measure impairment of social interaction, limitation of communication, and presence of repetitive and stereotypic behaviors) and cholesterol levels, there was a significant average decrease in the level of cholesterol within the red blood cell membranes sampled from children with ASD. However, some of the cholesterol levels were within the range considered to be normal, supporting previous findings [Tierney et al., 2006] suggesting that only about one in five children with ASD exhibit a significant decreased level of serum cholesterol levels compared to controls.

Pearson [2010] did not find a significant difference in cholesterol levels, 7DHC, or mutations in the DHCR7 gene between individuals in a community clinic for individuals with ASD (*n* = 42) and a control population (*n* = 27) [Pearson, 2010]. However, mutations in the gene DHCR24, a cholesterol synthesis gene, have been reported to be associated with ASD [Netting, 2013]. There have been a number of other studies which have found abnormally high total cholesterol levels and LDL cholesterol in individuals with ASD [e.g., Dziobek, Gold, Wolf, & Convit, 2007] and in patients with Rett syndrome [e.g., Segatto et al., 2014]. Consistent with this, statins (inhibitors of cholesterol biosynthesis) ameliorate the systemic imbalance of lipid profile, alleviate motor symptoms, and confer increased longevity in a mouse model of Rett syndrome [Buchovecky et al., 2013]. Additionally, recently Blassberg et al. found results which indicated that reduced levels of cholesterol impairs smoothened activation in SLOS [Blassberg, Macrae, Briscoe, & Jacob, 2016]. [“Smoothened (SMO)” is a protein involved in transmembrane signaling during the process of CNS formation]. Further study is required to elucidate this range of findings regarding the relationship between ASD and cholesterol. There are two lines of enquiry that look particularly promising. Specifically, studies investigating the supply of cholesterol sulfate to the fetus during gestation and the infant postnatally and studies examining myelination in the brains of individuals with ASD and cholesterol metabolic abnormalities.

### Cholesterol Sulfate Supply to the Fetus

An insufficient supply of cholesterol sulfate to the fetus during gestation and the infant postnatally has recently been postulated to be the primary cause of ASD. Seneff, Davidson, and Mascitelli [2012] hypothesized that the key factors, in both the affected child and the mother, are insufficient dietary sulfur and insufficient exposure to the sun [Seneff et al., 2012]. The exact mechanisms underlying how the mother supplies cholesterol to the fetus are unclear and merit more investigation given the crucial role of cholesterol in the development of the fetal nervous system. Cholesterol has been found to cross the blood brain barrier up to the 10th week of life. After that the fetus is dependent on its own production. Thus, mothers with high cholesterol levels can give birth to children with low cholesterol levels. Seneff, Davidson, Lauritzen, Samsel, and Wainwright [2015] have published a number of articles with hypotheses as to the role of cholesterol sulfate and sulfur such as a hypothesis for atherosclerosis as a cholesterol sulfate deficiency syndrome.

In sum, research to date provides some support for the hypothesis that defective cholesterol and related compound homeostasis may be responsible for the onset of ASD in some children, suggesting that investigating whether cholesterol may be a useful biomarker for certain ASD subtypes is worthwhile [Woods et al., 2013].

### Myelination in the Brains of Individuals with ASD and Cholesterol Metabolic Abnormalities

The largest pool of cholesterol exists in myelin membranes and disorders that interfere with sterol and cholesterol synthesis cause hypomyelination [Saher & Stumpf, 2015]. In addition, myelination abnormalities have been detected in patients with Fragile X syndrome suggesting that dysfunctions of myelination at the cellular and/or circuitry level may play a role in the pathological manifestations of this syndrome [Kalinowska, Castillo, & Francesconi, 2015]. Also, in autism in general, abnormalities in white matter integrity and in functional connectivity have been demonstrated [Kana, Libero, Hu, Deshpande, & Colburn, 2014; Travers et al., 2012] supporting the interdependence of sterol/lipid metabolism and autism.

## Future Directions

This hypothesis paper highlights the need for future studies to investigate the levels of multiple steroid hormones in individuals with ASD – especially cholesterol and VitD. To further explore the role of cholesterol in children with ASD, it might be possible to analyse the neonatally taken Guthrie spots with regard to relevant compounds [Starck & Lövgren, 2000], based on access to representative large clinical samples of children with ASD and controls. Lastly, SLOS patients can, theoretically, have adequate levels of VitD from either sun exposure or supplementation because the pathways for VitD and cholesterol synthesis are completely separated. Therefore, low levels of cholesterol do not imply low VitD. Future study could investigate VitD levels in individuals with SLOS.

The study carried out by Baron‐Cohen et al. [2015], reviewed above in the “Testosterone in Autism Spectrum Disorders” subsection, was the first direct evidence of elevated fetal steroidogenic activity in ASD. Their findings are consistent with the fetal steroid theory in providing some understanding as to some features of early epigenetic risk for ASD and/or other sex‐biased neurodevelopmental conditions which occur during fetal brain development. They argued that these elevated levels of fetal steroidogenic activity may be “important as epigenetic fetal programming mechanisms and may interact with other important pathophysiological factors in autism” [Baron‐Cohen et al., 2015, p. 370] and highlighted the need for future research to explore the potential underlying mechanisms, as well as the role of sex steroids, in the etiology of ASD [Baron‐Cohen et al., 2015]. We concur, given the findings across the studies reported in the present review, that there is a significant important need to investigate this area of research in more detail. Moreover, Baron‐Cohen et al. [2015] also point out a potential limitation with their study in that they did not test the source of elevated steroidogenic activity in the fetal development of ASD. As a result, they argue that there is a requirement for more research in order to understand the way in which a variety of sources (including, the fetus, mother, placenta, or other environmental factors) might contribute to the observed elevated concentration levels of Δ4 sex steroids (progesterone, 17α‐hydroxy‐progesterone, androstenedione, and testosterone) and cortisol that they found in their study. Further, Baron‐Cohen et al. argued that it is important for future research to investigate if “such elevations are specific to ASD or are shared by other neurodevelopmental conditions with skewed sex ratios, and to test whether similar elevations exist in females with autism” [Baron‐Cohen et al., 2015, p. 379]. Given the well‐established co‐morbidity between ASD and other sex‐biased conditions including ADHD, conduct disorder (CD) and anxiety disorder as well as the relationships between these sex‐biased conditions and levels of prenatal stress [e.g., O'Connor, Heron, Golding, & Glover, 2003; Simonoff et al., 2008], the elevated levels of fetal steroid activity may not be unique to ASD [Baron‐Cohen et al., 2015].

Additionally, the study carried out by Kosidou et al. [2016] (and reviewed in the same subsection as the study by Baron‐Cohen et al. [2015] discussed above) found that children of mothers with PCOS are at increased risk of developing ASD. The risk was the same irrespective of sex. These findings are consistent with the theory that in both males and females, early life androgen exposure may play a crucial role in the development of ASD. Therefore, research investigating the mechanisms involving sex steroids in the etiology of ASD is needed [Kosidou et al., 2016]. Although there are numerous studies suggesting that there is an interaction between genetic and environmental factors in the etiology of ASD, our relatively sparse knowledge and understanding of the underlying mechanisms presents potential barriers to informing the development of appropriate, timely and effective ways of identifying and preventing ASD [Kosidou et al., 2016]. Recently, Whitehouse [2016] argued that “future research must first understand how the prenatal hormone environment relates to individual behavioral dimensions, and then incorporate this knowledge into the investigation of links with the more aetiologically and phenotypically complex profile of ASD” [Whitehouse, 2016, p. 1,464].

We would suggest that in new studies in the field of neurodevelopmental disorders and possible links with sterol abnormalities, all of the following (and possibly more) should be included in the “work‐up kit” in all individuals recruited for study: serum levels of cholesterol, 7‐ and 8 dehydrocholesterol, 25‐OH vitamin D, testosterone, estrogen, cortisol (although diurnal variation may be a particular aspect to consider here) and 7‐dehydrocholesterol reductase. In collaboration with chemists and geneticists a variety of enzyme and gene tests may then be added to the protocol depending on specific research questions in any particular study.

## Conclusions

The “extreme male brain” theory of ASD [Baron‐Cohen, 2002] has generated hypotheses about the role of elevated fetal sex steroids such as testosterone in the development of ASD. However, it does not discuss the contribution of other factors such as sex chromosome effects or the involvement of other steroid hormones proximal to testosterone in biosynthesis pathways including: VitD, estradiol, cortisol, and progesterone. So, although the majority of research to date has focused on fetal testosterone and ASD, it appears that the actions of testosterone as well as other precursor steroid hormones within the same biosynthesis pathway may all contribute to fetal development in ASD [Baron‐Cohen et al., 2015].

In this hypothesis paper, we reviewed studies which linked some of the steroid hormones, specifically VitD, with levels of cholesterol. The steroid hormones we reviewed (testosterone; estrogens; cortisol, and VitD) all have studies which indicate their link with ASD. Therefore, it is possible that there may exist a cholesterol‐steroid hormone pathway underlying the development of ASD.

In sum, this review indicates that there may be links between “steroid metabolism” and findings of steroid abnormalities of various kinds (cortisol, testosterone, estrogens, vitamin D) in ASD. Further research investigating these potential links is warranted to further our understanding of the biological mechanisms underlying ASD.

## References

[aur1777-bib-0001] Al‐Ayadhi, L.Y. (2005). Altered oxytocin and vasopressin levels in autistic children in Central Saudi Arabia. Neurosciences, 10, 47–50. 22473184

[aur1777-bib-0002] Alexander, S. , Mathie, A. , & Peters, J. (2011). G protein‐coupled receptors. British Journal of Pharmacology, 164, S5–S113. doi:10.1111/j.1476-5381.2011.01649_3.x 10.1111/j.1476-5381.2011.01649_1.x PMC3315626 22040146

[aur1777-bib-0194] American Psychiatric Association . (2013). Diagnostic and statistical manual of mental disorders (5th ed.) Arlington, VA: American Psychiatric Publishing.

[aur1777-bib-0003] Anagnostou, E. , Soorya, L. , Chaplin, W. , Bartz, J. , Halpern, D. , Wasserman, S. , … Hollander, E. (2012). Intranasal oxytocin versus placebo in the treatment of adults with autism spectrum disorders: A randomized controlled trial. Molecular Autism, 3, 16. 23216716 10.1186/2040-2392-3-16 PMC3539865

[aur1777-bib-0004] Andari, E. , Duhamel, J.R. , Zalla, T. , Herbrecht, E. , Leboyer, M. , & Sirigu, A. (2010). Promoting social behavior with oxytocin in high‐functioning autism spectrum disorders. Proceedings of the National Academy of Sciences of the United States of America, 107, 4389–4394. 20160081 10.1073/pnas.0910249107 PMC2840168

[aur1777-bib-0005] Aneja, A. , & Tierney, E. (2008). Autism: The role of cholesterol in treatment. International Review of Psychiatry, 20, 165–170. 18386207 10.1080/09540260801889062

[aur1777-bib-0006] Angelopoulou, R. , Lavranos, G. , & Manolakou, P. (2006). Establishing sexual dimorphism in human. Collegium Antropologicum, 30, 653–658. 17058539

[aur1777-bib-0007] Asano, E. , Chugani, D.C. , Muzik, O. , Behen, M. , Janisse, J. , Rothermel, R. , … Chugani, H.T. (2001). Autism in tuberous sclerosis complex is related to both cortical and subcortical dysfunction. Neurology, 57, 1269–1277. 11591847 10.1212/wnl.57.7.1269

[aur1777-bib-0008] Asperger, H. (1991 ). ‘Autistic psychopathy’ in childhood (trans. and annotated by U. Firth) In U. Firth (Ed.), Autism and Asperger's syndrome (pp. 37–92). England: Cambridge University Press.

[aur1777-bib-0009] Auyeung, B. , & Baron‐Cohen, S. (2013). Fetal testosterone in mind: Implications for autism In Pfaff D.W., Christen Y. (Eds.) Multiple origins of sex differences in brain (pp. 123–137). Germany: Springer Berlin Heidelberg.

[aur1777-bib-0195] Auyeung, B. , Baron‐Cohen, S. , Ashwin, E. , Knickmeyer, R. , Taylor, K. , & Hackett, G. (2009). Fetal testosterone and autistic traits. British Journal of Psychology, 100, 1–22. 18547459 10.1348/000712608X311731

[aur1777-bib-0010] Auyeung, B. , Taylor, K. , Hackett, G. , & Baron‐Cohen, S. (2010). Research Foetal testosterone and autistic traits in 18 to 24‐month‐old children. Molecular Autism, 1, 1–8. 20678186 10.1186/2040-2392-1-11 PMC2916006

[aur1777-bib-0011] Azzam, H.M. , Sayyah, H. , Youssef, S. , Lotfy, H. , Abdelhamid, I.A. , Elhamed, H.A.A. , & Maher, S. (2015). Autism and vitamin D: An intervention study. Middle East Current Psychiatry, 22, 9–14.

[aur1777-bib-0012] Baieli, S. , Pavone, L. , Meli, C. , Fiumara, A. , & Coleman, M. (2003). Autism and phenylketonuria. Journal of Autism and Developmental Disorders, 33, 201–204. 12757360 10.1023/a:1022999712639

[aur1777-bib-0013] Barnevik‐Olsson, M. , Gillberg, C. , & Fernell, E. (2008). Prevalence of autism in children born to Somali parents living in Sweden: A brief report. Developmental Medicine and Child Neurology, 50, 598–601. 18754897 10.1111/j.1469-8749.2008.03036.x

[aur1777-bib-0014] Baron‐Cohen, S. (2002). The extreme male brain theory of autism. Trends in Cognitive Sciences, 6, 248–254. 12039606 10.1016/s1364-6613(02)01904-6

[aur1777-bib-0015] Baron‐Cohen, S. (2005). Testing the extreme male brain (EMB) theory of autism: Let the data speak for themselves. Cognitive Neuropsychiatry, 10, 77–81. 16571453 10.1080/13546800344000336

[aur1777-bib-0016] Baron‐Cohen, S. , Auyeung, B. , Nørgaard‐Pedersen, B. , Hougaard, D.M. , Abdallah, M.W. , Melgaard, L. , … Lombardo, M.V. (2015). Elevated fetal steroidogenic activity in autism. Molecular Psychiatry, 20, 369–376. 24888361 10.1038/mp.2014.48 PMC4184868

[aur1777-bib-0018] Bejerot, S. , & Eriksson, J.M. (2014). Sexuality and gender role in autism spectrum disorder: A case control study. PloS One, 9, e87961. 24498228 10.1371/journal.pone.0087961 PMC3909328

[aur1777-bib-0193] Bejerot, S. , Eriksson, J. M. , Bonde, S. , Carlström, K. , Humble, M. B. , & Eriksson, E. (2012). The extreme male brain revisited: gender coherence in adults with autism spectrum disorder. The British Journal of Psychiatry, 201, 116–123. 22500012 10.1192/bjp.bp.111.097899

[aur1777-bib-0019] Bener, A. , Khattab, A.O. , & Al‐Dabbagh, M.M. (2014). Is high prevalence of Vitamin D deficiency evidence for autism disorder? In a highly endogamous population. Journal of Pediatric Neurosciences, 9, 227. 25624924 10.4103/1817-1745.147574 PMC4302541

[aur1777-bib-0020] Berry‐Kravis, E. , Levin, R. , Shah, H. , Mathur, S. , Darnell, J.C. , & Ouyang, B. (2015). Cholesterol levels in Fragile X syndrome. American Journal of Medical Genetics Part A, 167, 379–384. 10.1002/ajmg.a.36850 PMC5436717 25424470

[aur1777-bib-0022] Bitsika, V. , Sharpley, C.F. , Sweeney, J.A. , & McFarlane, J.R. (2014). HPA and SAM axis responses as correlates of self‐vs parental ratings of anxiety in boys with an Autistic Disorder. Physiology & Behavior, 127, 1–7. 24412722 10.1016/j.physbeh.2013.12.011

[aur1777-bib-0023] Blassberg, R. , Macrae, J.I. , Briscoe, J. , & Jacob, J. (2016). Reduced cholesterol levels impair Smoothened activation in Smith–Lemli–Opitz syndrome. Human Molecular Genetics, 25(4), 693–705. 26685159 10.1093/hmg/ddv507 PMC4743690

[aur1777-bib-0024] Bolton, P.F. , & Griffiths, P.D. (1997). Association of tuberous sclerosis of temporal lobes with autism and atypical autism. The Lancet, 349, 392–395. 10.1016/S0140-6736(97)80012-8 9033466

[aur1777-bib-0025] Brosnan, M. , Turner‐Cobb, J. , Munro‐Naan, Z. , & Jessop, D. (2009). Absence of a normal cortisol awakening response (CAR) in adolescent males with Asperger syndrome (AS). Psychoneuroendocrinology, 34, 1095–1100. 19304400 10.1016/j.psyneuen.2009.02.011

[aur1777-bib-0026] Brown, W.T. , Friedman, E. , Jenkins, E.C. , Brooks, J. , Wisniewski, K. , Raguthu, S. , & French, J.H. (1982). Association of fragile X syndrome with autism. The Lancet, 319, 100. 10.1016/s0140-6736(82)90231-8 6119460

[aur1777-bib-0028] Bukelis, I. , Porter, F.D. , Zimmerman, A.W. , & Tierney, E. (2007). Smith‐Lemli‐Opitz syndrome and autism spectrum disorder. American Journal of Psychiatry, 164, 1655–1661. 17974928 10.1176/appi.ajp.2007.07020315

[aur1777-bib-0030] Cannell, J.J. (2008). Autism and vitamin D. Medical Hypotheses, 70, 750–759. 17920208 10.1016/j.mehy.2007.08.016

[aur1777-bib-0031] Cannell, J.J. (2013). Autism, will vitamin D treat core symptoms? Medical Hypotheses, 81, 195–198. 23725905 10.1016/j.mehy.2013.05.004

[aur1777-bib-0032] Cannell, J.J. , & Grant, W.B. (2012). What is the role of vitamin D in autism? Dermato‐Endocrinology, 5, 199–204. 10.4161/derm.24356 PMC3897590 24494055

[aur1777-bib-0033] Chakrabarti, B. , Dudbridge, F. , Kent, L. , Wheelwright, S. , Hill‐Cawthorne, G. , Allison, C. , … & Baron‐Cohen, S. (2009). Genes related to sex steroids, neural growth, and social‐emotional behavior are associated with autistic traits, empathy, and Asperger syndrome. Autism Research, 2, 157–177. 19598235 10.1002/aur.80

[aur1777-bib-0034] Christodoulou, S. , Goula, T. , Ververidis, A. , & Drosos, G. (2013). Vitamin D and bone disease. BioMed Research International, 2013, 396541. 23509720 10.1155/2013/396541 PMC3591184

[aur1777-bib-0035] Clemens, T.L. , Adams, J.S. , Henderson, S.L. , & Holick, M.F. (1982). Increased skin pigment reduces the capacity of skin to synthesise vitamin D3. Lancet, 1, 74–76. 6119494 10.1016/s0140-6736(82)90214-8

[aur1777-bib-0036] Coleman, M. , & Gillberg, C. (2012). The autisms (4th ed). New York: Oxford University Press.

[aur1777-bib-0038] Corbett, B.A. , & Schupp, C.W. (2014). The cortisol awakening response (CAR) in male children with autism spectrum disorder. Hormones and Behavior, 65, 345–350. 24508619 10.1016/j.yhbeh.2014.01.012 PMC4004674

[aur1777-bib-0039] Corbett, B.A. , & Simon, D. (2013). Adolescence, stress and cortisol in autism spectrum disorders. OA Autism, 1, 2. PMC3961758 24665363

[aur1777-bib-0040] Corbett, B.A. , Mendoza, S. , Abdullah, M. , Wegelin, J.A. , & Levine, S. (2006). Cortisol circadian rhythms and response to stress in children with autism. Psychoneuroendocrinology, 31, 59–68. 16005570 10.1016/j.psyneuen.2005.05.011

[aur1777-bib-0041] Corbett, B.A. , Mendoza, S. , Wegelin, J.A. , Carmean, V. , & Levine, S. (2008). Variable cortisol circadian rhythms in children with autism and anticipatory stress. Journal of Psychiatry and Neuroscience, 33, 227–234. 18592041 PMC2441887

[aur1777-bib-0042] Corbett, B.A. , Schupp, C.W. , & Lanni, K.E. (2012). Comparing biobehavioral profiles across two social stress paradigms in children with and without autism spectrum disorders. Molecular Autism, 3, 1–10. 23158965 10.1186/2040-2392-3-13 PMC3533919

[aur1777-bib-0043] Corbett, B.A. , Schupp, C.W. , Levine, S. , & Mendoza, S. (2009). Comparing cortisol, stress and sensory sensitivity in children with autism. Autism Research, 2, 39–49. 19358306 10.1002/aur.64 PMC2698454

[aur1777-bib-0044] Crespi, B.J. , & Hurd, P.L. (2015). Genetically based correlates of serum oxytocin and testosterone in autism and schizotypy. Personality and Individual Differences, 79, 39–43.

[aur1777-bib-0045] Crider, A. , Thakkar, R. , Ahmed, A.O. , & Pillai, A. (2014). Dysregulation of estrogen receptor beta (ERβ), aromatase (CYP19A1), and ER co‐activators in the middle frontal gyrus of autism spectrum disorder subjects. Molecular Autism, 5, 46. 25221668 10.1186/2040-2392-5-46 PMC4161836

[aur1777-bib-0046] De Souza Tostes, M.H. , Polonini, H.C. , Gattaz, W.F. , Raposo, N.R. , & Baptista, E.B. (2012). Low serum levels of 25‐hydroxyvitamin D (25‐OHD) in children with autism. Trends in Psychiatry and Psychotherapy, 34, 161–163. 25923008 10.1590/s2237-60892012000300008

[aur1777-bib-0047] De Vries, A.L. , Noens, I.L. , Cohen‐Kettenis, P.T. , van Berckelaer‐Onnes, I.A. , & Doreleijers, T.A. (2010). Autism spectrum disorders in gender dysphoric children and adolescents. Journal of Autism and Developmental Disorders, 40, 930–936. 20094764 10.1007/s10803-010-0935-9 PMC2904453

[aur1777-bib-0048] Dealberto, M.J. (2011). Prevalence of autism according to maternal immigrant status and ethnic origin. Acta Psychiatrica Scandinavica, 123, 339–348. 21219265 10.1111/j.1600-0447.2010.01662.x

[aur1777-bib-0050] Diaz‐Stransky, A. , & Tierney, E. (2014). Cholesterol use in autism treatment. In comprehensive guide to autism (pp. 2403–2425). New York: Springer.

[aur1777-bib-0051] Domes, G. , Heinrichs, M. , Kumbier, E. , Grossmann, A. , Hauenstein, K. , & Herpertz, S.C. (2013). Effects of intranasal oxytocin on the neural basis of face processing in autism spectrum disorder. Biological Psychiatry, 74, 164–171. 23510581 10.1016/j.biopsych.2013.02.007

[aur1777-bib-0052] Durdiakova, J. , Ostatnikova, D. , & Celec, P. (2011). Testosterone and its metabolites–modulators of brain functions. Acta Neurobiologiae Experimentalis, 71, 434–454. 22237492 10.55782/ane-2011-1863

[aur1777-bib-0053] Dziobek, I. , Gold, S.M. , Wolf, O.T. , & Convit, A. (2007). Hypercholesterolemia in Asperger syndrome: Independence from lifestyle, obsessive–compulsive behavior, and social anxiety. Psychiatry Research, 149, 321–324. 17123635 10.1016/j.psychres.2006.02.003

[aur1777-bib-0054] Ehrhardt, A.A. , Meyer‐Bahlburg, H.F. , Rosen, L.R. , Feldman, J.F. , Veridiano, N.P. , Zimmerman, I. , & McEwen, B.S. (1985). Sexual orientation after prenatal exposure to exogenous estrogen. Archives of Sexual Behavior, 14, 57–77. 3977584 10.1007/BF01541353

[aur1777-bib-0055] Fein, D. , Joy, S. , Green, L. , & Waterhouse, L. (1996). Autism and pervasive developmental disorders In Fogel B., Schiffer R., Rao S. (Eds.), Neuropsychiatry. Baltimore, MD: Williams & Wilkins.

[aur1777-bib-0057] Fernell, E. , Bejerot, S. , Westerlund, J. , Miniscalco, C. , Simila, H. , Eyles, D. , … Humble, M.B. (2015). Autism spectrum disorder and low vitamin D at birth: A sibling control study. Molecular Autism, 6, 3. 25874075 10.1186/2040-2392-6-3 PMC4396835

[aur1777-bib-0059] Fierro, M. , Martinez, A.J. , Harbison, J.W. , & Hay, S.H. (1977). Smith‐Lemli‐Opitz syndrome: Neuropathological and ophthalmological observations. Developmental Medicine and Child Neurology, 19, 57–62. 844667 10.1111/j.1469-8749.1977.tb08021.x

[aur1777-bib-0060] Gainer, H. , Altstein, M. , & Whitnall, M.H. (1987). The cell biology and development of vasopressinergic and oxytocinergic neurons. Progress in Brain Research, 72, 153–161. 3039572 10.1016/s0079-6123(08)60204-6

[aur1777-bib-0196] Gillberg, C. (1991). Debate and argument: is autism a pervasive developmental disorder?. Journal of Child Psychology and Psychiatry, 32, 1169–1170. 1787143 10.1111/j.1469-7610.1991.tb00357.x

[aur1777-bib-0067] Gillberg, I.C. , Gillberg, C. , & Ahlsén, G. (1994). Autistic behaviour and attention deficits in tuberous sclerosis: A population‐based study. Developmental Medicine & Child Neurology, 36, 50–56. 8132114 10.1111/j.1469-8749.1994.tb11765.x

[aur1777-bib-0069] Gillberg, C. , Schaumann, H. , & Gillberg, I.C. (1995). Autism in immigrants: Children born in Sweden to mothers born in Uganda. Journal of Intellectual Disability Research, 39, 141–144. 7787384 10.1111/j.1365-2788.1995.tb00482.x

[aur1777-bib-0070] Gimpl, G. , & Fahrenholz, F. (2001). The oxytocin receptor system: Structure, function, and regulation. Physiological Reviews, 81, 629–683. 11274341 10.1152/physrev.2001.81.2.629

[aur1777-bib-0071] Gimpl, G. , Reitz, J. , Brauer, S. , & Trossen, C. (2008). Oxytocin receptors: Ligand binding, signalling and cholesterol dependence. Progress in Brain Research, 170, 193–204. 18655883 10.1016/S0079-6123(08)00417-2

[aur1777-bib-0072] Gong, Z.L. , Luo, C.M. , Wang, L. , Shen, L. , Wei, F. , Tong, R.J. , & Liu, Y. (2014). Serum 25‐hydroxyvitamin D levels in Chinese children with autism spectrum disorders. Neuroreport, 25, 23–27. 24089013 10.1097/WNR.0000000000000034

[aur1777-bib-0073] Goodman, R. , & Richards, H. (1995). Child and adolescent psychiatric presentations of second‐generation Afro‐Caribbeans in Britain. British Journal of Psychiatry, 167, 362–369. 7496645 10.1192/bjp.167.3.362

[aur1777-bib-0075] Grant, W.B. , & Cannell, J.J. (2013). Autism prevalence in the United States with respect to solar UV‐B doses: An ecological study. Dermato‐Endocrinology, 5, 159–164. 24494049 10.4161/derm.22942 PMC3897584

[aur1777-bib-0076] Grant, W.B. , & Soles, C.M. (2009). Epidemiologic evidence supporting the role of maternal vitamin D deficiency as a risk factor for the development of infantile autism. Dermato‐Endocrinology, 1, 223–228. 20592795 10.4161/derm.1.4.9500 PMC2835879

[aur1777-bib-0077] Green, L. , Fein, D. , Modahl, C. , Feinstein, C. , Waterhouse, L. , & Morris, M. (2001). Oxytocin and autistic disorder: Alterations in peptide forms. Biological Psychiatry, 50, 609–613. 11690596 10.1016/s0006-3223(01)01139-8

[aur1777-bib-0199] Green, J. J. , & Hollander, E. (2010). Autism and oxytocin: new developments in translational approaches to therapeutics. Neurotherapeutics, 7, 250–257. 20643377 10.1016/j.nurt.2010.05.006 PMC5084228

[aur1777-bib-0078] Guastella, A.J. , Einfeld, S.L. , Gray, K.M. , Rinehart, N.J. , Tonge, B.J. , Lambert, T.J. , & Hickie, I.B. (2010). Intranasal oxytocin improves emotion recognition for youth with autism spectrum disorders. Biological Psychiatry, 67, 692–694. 19897177 10.1016/j.biopsych.2009.09.020

[aur1777-bib-0080] Hediger, M.L. , England, L.J. , Molloy, C.A. , Kai, F.Y. , Manning‐Courtney, P. , & Mills, J.L. (2008). Reduced bone cortical thickness in boys with autism or autism spectrum disorder. Journal of Autism and Developmental Disorders, 38, 848–856. 17879151 10.1007/s10803-007-0453-6

[aur1777-bib-0081] Hennessey, J.W. , & Levine, S. (1979). Stress, arousal, and the pituitary‐adrenal system: A psychoendocrine hypothesis. In Sprague J.M. & Epstein A.N. (Eds.), New York: Academic Press.

[aur1777-bib-0082] Herbert, M.R. (2010). Contributions of the environment and environmentally vulnerable physiology to autism spectrum disorders. Current Opinion in Neurology, 23, 103–110. 20087183 10.1097/WCO.0b013e328336a01f

[aur1777-bib-0083] Herman, J.P. , & Cullinan, W.E. (1997). Neurocircuitry of stress: Central control of the hypothalamo–pituitary–adrenocortical axis. Trends in Neurosciences, 20, 78–84. 9023876 10.1016/s0166-2236(96)10069-2

[aur1777-bib-0084] Holick, M.F. (2007). Medical progress: Vitamin D deficiency. The New England Journal of Medicine, 357, 266–281. 17634462 10.1056/NEJMra070553

[aur1777-bib-0085] Hollander, E. (2003). Oxytocin infusion reduces repetitive behaviors in adults withautistic and Asperger's disorders. Neuropsychopharmacology, 28, 193–198. 12496956 10.1038/sj.npp.1300021

[aur1777-bib-0086] Hollander, E. , Bartz, J. , Chaplin, W. , Phillips, A. , Sumner, J. , Soorya, L. , … Wasserman, S. (2007). Oxytocin increases retention of social cognition in autism. Biological Psychiatry, 61, 498–503. 16904652 10.1016/j.biopsych.2006.05.030

[aur1777-bib-0087] Humble, M.B. , Gustafsson, S. , & Bejerot, S. (2010). Low serum levels of 25‐hydroxyvitamin D (25‐OHD) among psychiatric out‐patients in Sweden: Relations with season, age, ethnic origin and psychiatric diagnosis. The Journal of Steroid Biochemistry and Molecular Biology, 121, 467–470. 20214992 10.1016/j.jsbmb.2010.03.013

[aur1777-bib-0197] Ingudomnukul, E. , Baron‐Cohen, S. , Wheelwright, S. , & Knickmeyer, R. (2007). Elevated rates of testosterone‐related disorders in women with autism spectrum conditions. Hormones and Behavior, 51, 597–604. 17462645 10.1016/j.yhbeh.2007.02.001

[aur1777-bib-0198] Jia, F. , Wang, B. , Shan, L. , Xu, Z. , Staal, W. G. , & Du, L. (2015). Core symptoms of autism improved after vitamin D supplementation. Pediatrics, 135, e196–e198. 25511123 10.1542/peds.2014-2121

[aur1777-bib-0088] Jiang, X.S. , Wassif, C.A. , Backlund, P.S. , Song, L. , Holtzclaw, L.A. , Li, Z. , Yergey, A.L. , & Porter, F.D. (2010). Activation of Rho GTPases in Smith–Lemli–Opitz syndrome: Pathophysiological and clinical implications. Human Molecular Genetics, 19, 1347–1357. 20067919 10.1093/hmg/ddq011 PMC2838542

[aur1777-bib-0089] Kalinowska, M. , Castillo, C. , & Francesconi, A. (2015). Quantitative profiling of brain lipid raft proteome in a mouse model of fragile X syndrome. PloS One, 10, e0121464. 25849048 10.1371/journal.pone.0121464 PMC4388542

[aur1777-bib-0090] Kana, R.K. , Libero, L.E. , Hu, C.P. , Deshpande, H.D. , & Colburn, J.S. (2014). Functional brain networks and white matter underlying theory‐of‐mind in autism. Social Cognitive and Affective Neuroscience, 9, 98–105. 22977198 10.1093/scan/nss106 PMC3871731

[aur1777-bib-0091] Kanner, L. (1943). Autistic disturbances of affective contact. Nervous Child, 2, 217–250. 4880460

[aur1777-bib-0092] Keen, D.V. , Reid, F.D. , & Arnone, D. (2010). Autism, ethnicity and maternal immigration. British Journal of Psychiatry, 196, 274–281. 20357302 10.1192/bjp.bp.109.065490

[aur1777-bib-0094] Kirkovski, M. , Enticott, P.G. , & Fitzgerald, P.B. (2013). A review of the role of female gender in autism spectrum disorders. Journal of Autism and Developmental Disorders, 43, 2584–2603. 23525974 10.1007/s10803-013-1811-1

[aur1777-bib-0096] Knickmeyer, R. , Baron‐Cohen, S. , Fane, B.A. , Wheelwright, S. , Mathews, G.A. , Conway, G.S. , … Hines, M. (2006). Androgens and autistic traits: A study of individuals with congenital adrenal hyperplasia. Hormones and Behavior, 50, 148–153. 16624315 10.1016/j.yhbeh.2006.02.006

[aur1777-bib-0104] Kočovská, E. , Andorsdóttir, G. , Weihe, P. , Halling, J. , Fernell, E. , Stóra, T. , … Gillberg, C. (2014). Vitamin D in the general population of young adults with autism in the Faroe Islands. Journal of Autism and Developmental Disorders, 44, 2996–3005. 24927807 10.1007/s10803-014-2155-1 PMC4221602

[aur1777-bib-0105] Kočovská, E. , Fernell, E. , Billstedt, E. , Minnis, H. , & Gillberg, C. (2012). Vitamin D and autism: Clinical review. Research in Developmental Disabilities, 33, 1541–1550. 22522213 10.1016/j.ridd.2012.02.015

[aur1777-bib-0106] Kosidou, K. , Dalman, C. , Widman, L. , Arver, S. , Lee, B.K. , Magnusson, C. , & Gardner, R.M. (2016). Maternal polycystic ovary syndrome and the risk of autism spectrum disorders in the offspring: A population‐based nationwide study in Sweden. Molecular Psychiatry, 21, 1441–1448. 26643539 10.1038/mp.2015.183 PMC5030459

[aur1777-bib-0108] Krajmer, P. , Jánošíková, D. , Špajdel, M. , & Ostatníková, D. (2010). Empathizing, systemizing, intuitive physics and folk psychology in boys with Asperger syndrome. Activitas Nervosa Superior Rediviva, 52, 57–61.

[aur1777-bib-0109] Kumar, V.S. (2014). Phenylketonuria with autism spectrum disorders: A case study. International Journal of Health Sciences and Research (IJHSR), 4, 308–312.

[aur1777-bib-0110] Lai, M.C. , Lerch, J.P. , Floris, D.L. , Ruigrok, A.N. , Pohl, A. , Lombardo, M.V. , & Baron ‐ Cohen, S. (2017). Imaging sex/gender and autism in the brain: Etiological implications. Journal of Neuroscience Research, 95, 380–397. 27870420 10.1002/jnr.23948

[aur1777-bib-0111] Lai, M.C. , Lombardo, M.V. , Suckling, J. , Ruigrok, A.N. , Chakrabarti, B. , Ecker, C. , … Baron‐Cohen, S. (2013). Biological sex affects the neurobiology of autism. Brain, 136, 2799–2815. 23935125 10.1093/brain/awt216 PMC3754459

[aur1777-bib-0112] Lakatosova, S. , Nurit, S. , Anna, P. , Veronika, H. , Irina, R. , Daniela, O. , & Maria, C.A. (2013). Oxytocin but not testosterone modulates behavioral patterns in autism spectrum disorders. Open Journal of Medical Psychology, 3, 48–53.

[aur1777-bib-0113] Landén, M. , Wålinder, J. , & Lundström, B. (1996). Prevalence, incidence and sex ratio of transsexualism. Acta Psychiatrica Scandinavica, 93, 221–223. 8712018 10.1111/j.1600-0447.1996.tb10638.x

[aur1777-bib-0114] Lecis, C. , & Segatto, M. (2014). Cholesterol homeostasis imbalance and brain functioning: Neuro‐logical disorders and behavioral consequences. Journal of Neurology and Neurological Disorders, 1, 1.

[aur1777-bib-0115] Lee, R.W. , & Tierney, E. (2011). Hypothesis: The role of sterols in autism spectrum disorder. Autism Research and Treatment, 7 p. 10.1155/2011/653570 PMC3420784 22937253

[aur1777-bib-0116] Leffell, D.J. , & Brash, D.E. (1996). Sunlight and skin cancer. Scientific American, 275, 52–59. 10.1038/scientificamerican0796-52 8658110

[aur1777-bib-0118] Liberati, A. , Altman, D.G. , Tetzlaff, J. , Mulrow, C. , Gøtzsche, P.C. , Ioannidis, J.P. , … Moher, D. (2009). The PRISMA statement for reporting systematic reviews and meta‐analyses of studies that evaluate health care interventions: Explanation and elaboration. Annals of Internal Medicine, 151, W‐65. 10.7326/0003-4819-151-4-200908180-00136 19622512

[aur1777-bib-0119] Lord, C. , Rutter, M. , & Le Couteur, A. (1994). Autism diagnostic interview‐revised: A revised version of a diagnostic interview for caregivers of individuals with possible pervasive developmental disorders. Journal of Autism and Developmental Disorders, 24, 659–685. 7814313 10.1007/BF02172145

[aur1777-bib-0120] Lydon, S. , Healy, O. , Roche, M. , Henry, R. , Mulhern, T. , & Hughes, B.M. (2015). Salivary cortisol levels and challenging behavior in children with autism spectrum disorder. Research in Autism Spectrum Disorders, 10, 78–92.

[aur1777-bib-0121] Marcos, J. , Guo, L.W. , Wilson, W.K. , Porter, F.D. , & Shackleton, C. (2004). The implications of 7‐dehydrosterol‐7‐reductase deficiency (Smith–Lemli–Opitz syndrome) to neurosteroid production. Steroids, 69, 51–60. 14715377 10.1016/j.steroids.2003.09.013

[aur1777-bib-0122] Meguid, N.A. , Hashish, A.F. , Anwar, M. , & Sidhom, G. (2010). Reduced serum levels of 25‐hydroxy and 1, 25‐dihydroxy vitamin D in Egyptian children with autism. The Journal of Alternative and Complementary Medicine, 16, 641–645. 20569030 10.1089/acm.2009.0349

[aur1777-bib-0123] Meljon, A. , Watson, G.L. , Wang, Y. , Shackleton, C.H. , & Griffiths, W.J. (2013). Analysis by liquid chromatography–mass spectrometry of sterols and oxysterols in brain of the newborn Dhcr7 Δ3‐5/T93M mouse: A model of Smith–Lemli–Opitz syndrome. Biochemical Pharmacology, 86, 43–55. 23500538 10.1016/j.bcp.2013.03.003 PMC4004445

[aur1777-bib-0124] Miller, M. , Bales, K.L. , Taylor, S.L. , Yoon, J. , Hostetler, C.M. , Carter, C.S. , & Solomon, M. (2013). Oxytocin and vasopressin in children and adolescents with autism spectrum disorders: Sex differences and associations with symptoms. Autism Research, 6, 91–102. 23413037 10.1002/aur.1270 PMC3657571

[aur1777-bib-0125] Modahl, C. , Fein, D. , Waterhouse, L. , & Newton, N. (1992). Does oxytocin deficiency mediate social deficits in autism? Journal of Autism and Developmental Disorders, 22, 449–451. 1400106 10.1007/BF01048246

[aur1777-bib-0126] Modahl, C. , Green, L.A. , Fein, D. , Morris, M. , Waterhouse, L. , Feinstein, C. , & Levin, H. (1998). Plasma oxytocin levels in autistic children. Biological Psychiatry, 43, 270–277. 9513736 10.1016/s0006-3223(97)00439-3

[aur1777-bib-0127] Moher, D. , Liberati, A. , Tetzlaff, J. , & Altman, D.G. (2009). Preferred reporting items for systematic reviews and meta‐analyses: The PRISMA statement. Annals of Internal Medicine, 151, 264–269. 19622511 10.7326/0003-4819-151-4-200908180-00135

[aur1777-bib-0128] Molloy, C.A. , Kalkwarf, H.J. , Manning‐Courtney, P. , Mills, J.L. , & Hediger, M.L. (2010). Plasma 25 (OH) D concentration in children with autism spectrum disorder. Developmental Medicine and Child Neurology, 52, 969–971. 20497452 10.1111/j.1469-8749.2010.03704.x PMC2939162

[aur1777-bib-0129] Mostafa, G.A. , & Al‐Ayadhi, L.Y. (2012). Reduced serum concentrations of 25‐hydroxy vitamin D in children with autism: Relation to autoimmunity. Journal of Neuroinflammation, 9, 201. 22898564 10.1186/1742-2094-9-201 PMC3476426

[aur1777-bib-0130] Muhle, R. , Trentacoste, S.V. , & Rapin, I. (2004). The genetics of autism. Pediatrics, 113, e472–e486. 15121991 10.1542/peds.113.5.e472

[aur1777-bib-0131] Netting, J. (2013). New autism gene plays key role in cholesterol synthesis. Spectrum. Accessed on 20 March 2016. Retrieved from https://spectrumnews.org/news/new-autism-gene-plays-key-role-in-cholesterol-synthesis/

[aur1777-bib-0132] Nishimori, K. , Takayanagi, Y. , Yoshida, M. , Kasahara, Y. , Young, L.J. , & Kawamata, M. (2008). New aspects of oxytocin receptor function revealed by knockout mice: Sociosexual behaviour and control of energy balance. Progress in Brain Research, 170, 79–90. 18655874 10.1016/S0079-6123(08)00408-1

[aur1777-bib-0133] O'Connor, T.G. , Heron, J. , Golding, J. , & Glover, V. (2003). Maternal antenatal anxiety and behavioural/emotional problems in children: A test of a programming hypothesis. Journal of Child Psychology and Psychiatry, 44, 1025–1036. 14531585 10.1111/1469-7610.00187

[aur1777-bib-0134] Opitz, J.M. , Gilbert‐Barness, E. , Ackerman, J. , & Lowichik, A. (2002). Cholesterol and development: The RSH (“ Smith‐Lemli‐Opitz”) syndrome and related conditions. Pediatric Pathology and Molecular Medicine, 21, 153–181. 11942534 10.1080/15227950252852078

[aur1777-bib-0135] Patrick, R.P. , & Ames, B.N. (2014). Vitamin D hormone regulates serotonin synthesis. Part 1: Relevance for autism. The FASEB Journal, 28, 2398–2413. 24558199 10.1096/fj.13-246546

[aur1777-bib-0136] Payne, A.H. , & Hales, D.B. (2004). Overview of steroidogenic enzymes in the pathway from cholesterol to active steroid hormones. Endocrine Reviews, 25, 947–970. 15583024 10.1210/er.2003-0030

[aur1777-bib-0137] Pearson, J. (2010). Disordered cholesterol metabolism in autism spectrum disorders sterol and genetic analyses. Scholar Archive, paper 437. Retrieved 23rd March 2016 from http://digitalcommons.ohsu.edu/cgi/viewcontent.cgi?article=1436&context=etd

[aur1777-bib-0138] Pfaff, D.W. , Rapin, I. , & Goldman, S. (2011). Male predominance in autism: Neuroendocrine influences on arousal and social anxiety. Autism Research, 4, 163–176. 21465671 10.1002/aur.191

[aur1777-bib-0139] Porter, F.D. (2008). Smith–Lemli–Opitz syndrome: Pathogenesis, diagnosis and management. European Journal of Human Genetics, 16, 535–541. 18285838 10.1038/ejhg.2008.10

[aur1777-bib-0140] Posserud, M.B. , Lundervold, A.J. , & Gillberg, C. (2006). Autistic features in a total population of 7–9‐year‐old children assessed by the ASSQ (Autism Spectrum Screening Questionnaire). Journal of Child Psychology and Psychiatry, 47, 167–175. 16423148 10.1111/j.1469-7610.2005.01462.x

[aur1777-bib-0141] Quattrocki, E. , & Friston, K. (2014). Autism, oxytocin and interoception. Neuroscience & Biobehavioral Reviews, 47, 410–430. 25277283 10.1016/j.neubiorev.2014.09.012 PMC4726659

[aur1777-bib-0142] Reversi, A. , Rimoldi, V. , Brambillasca, S. , & Chini, B. (2006). Effects of cholesterol manipulation on the signaling of the human oxytocin receptor. American Journal of. Physiology‐Regulatory, Integrative and Comparative Physiology, 291, R861–R869. 10.1152/ajpregu.00333.2006 16966388

[aur1777-bib-0143] Richdale, A.L. , & Prior, M.R. (1992). Urinary cortisol circadian rhythm in a group of high‐functioning children with autism. Journal of Autism and Developmental Disorders, 22, 433–447. 1400105 10.1007/BF01048245

[aur1777-bib-0144] Saad, K. , Abdel‐Rahman, A.A. , Elserogy, Y.M. , Al‐Atram, A.A. , John, J. , Cannell4, Geir Bjørklund, G. , … Ali, A.M. (2015). Vitamin D status in autism spectrum disorders and the efficacy of vitamin D supplementation in autistic children. Nutritional Neuroscience, 19, 346–351. 25876214 10.1179/1476830515Y.0000000019

[aur1777-bib-0145] Saad, K. , Hammad, E. , Abdel‐Rahman, A.A. , & Sobhy, K.M. (2013). Autistic symptoms in late diagnosed phenylketonuric children in Upper Egypt. Journal of Neurology Research, 3, 122–129.

[aur1777-bib-0146] Saher, G. , & Stumpf, S.K. (2015). Cholesterol in myelin biogenesis and hypomyelinating disorders. Biochimica Et Biophysica Acta (BBA)‐Molecular and Cell Biology of Lipids, 1851, 1083–1094. 25724171 10.1016/j.bbalip.2015.02.010

[aur1777-bib-0147] Schengrund, C.L. , Ali‐Rahmani, F. , & Ramer, J.C. (2012). Cholesterol, GM1, and autism. Neurochemical Research, 37, 1201–1207. 22252726 10.1007/s11064-011-0697-6

[aur1777-bib-0148] Schmidt, R.J. , Hansen, R.L. , Hartiala, J. , Allayee, H. , Sconberge, J.L. , Schmidt, L.C. , Volk, H.E. , & Tassone, F. (2015). Selected vitamin D metabolic gene variants and risk for autism spectrum disorder in the CHARGE Study. Early Human Development, 91, 483–489. 26073892 10.1016/j.earlhumdev.2015.05.008 PMC4871694

[aur1777-bib-0150] Segatto, M. , Trapani, L. , Di Tunno, I. , Sticozzi, C. , Valacchi, G. , Hayek, J. , & Pallottini, V. (2014). Cholesterol metabolism is altered in Rett syndrome: A study on plasma and primary cultured fibroblasts derived from patients. PLoS ONE, 9, e104834. 25118178 10.1371/journal.pone.0104834 PMC4130597

[aur1777-bib-0151] Seneff, S. , Davidson, R. , & Mascitelli, L. (2012). Might cholesterol sulfate deficiency contribute to the development of autistic spectrum disorder? Medical Hypotheses, 78, 213–217. 22098722 10.1016/j.mehy.2011.10.026

[aur1777-bib-0152] Seneff, S. , Davidson, R.M. , Lauritzen, A. , Samsel, A. , & Wainwright, G. (2015). A novel hypothesis for atherosclerosis as a cholesterol sulfate deficiency syndrome. Theoretical Biology and Medical Modelling, 12, 24. 26014131 10.1186/s12976-015-0006-1 PMC4456713

[aur1777-bib-0153] Sikora, D.M. , Pettit‐Kekel, K. , Penfield, J. , Merkens, L.S. , & Steiner, R.D. (2006). The near universal presence of autism spectrum disorders in children with Smith–Lemli–Opitz syndrome. American Journal of Medical Genetics Part A, 140, 1511–1518. 16761297 10.1002/ajmg.a.31294

[aur1777-bib-0154] Simonoff, E. , Pickles, A. , Charman, T. , Chandler, S. , Loucas, T. , & Baird, G. (2008). Psychiatric disorders in children with autism spectrum disorders: Prevalence, comorbidity, and associated factors in a population‐derived sample. Journal of the American Academy of Child and Adolescent Psychiatry, 47, 921–929. 18645422 10.1097/CHI.0b013e318179964f

[aur1777-bib-0155] Smalley, S.L. (1998). Autism and tuberous sclerosis. Journal of Autism and Developmental Disorders, 28, 407–414. 9813776 10.1023/a:1026052421693

[aur1777-bib-0157] Sparks, S.E. , Wassif, C.A. , Goodwin, H. , Conley, S.K. , Lanham, D.C. , Kratz, L.E. , … Porter, F.D. (2014). Decreased cerebral spinal fluid neurotransmitter levels in Smith‐Lemli‐Opitz syndrome. Journal of Inherited Metabolic Disease, 37, 415–420. 24500076 10.1007/s10545-013-9672-5 PMC4166510

[aur1777-bib-0158] Starck, L. , & Lövgren, A. (2000). Diagnosis of Smith‐Lemli‐Opitz syndrome from stored filter paper blood specimens. Archives of Disease in Childhood, 82, 490–492. 10833186 10.1136/adc.82.6.490 PMC1718355

[aur1777-bib-0159] Strang, J.F. , Kenworthy, L. , Dominska, A. , Sokoloff, J. , Kenealy, L.E. , Berl, M. , … Wallace, G.L. (2014). Increased gender variance in autism spectrum disorders and attention deficit hyperactivity disorder. Archives of Sexual Behavior, 43, 1525–1533. 24619651 10.1007/s10508-014-0285-3

[aur1777-bib-0160] Strifert, K. (2014). The link between oral contraceptive use and prevalence in autism spectrum disorder. Medical Hypotheses, 83, 718–725. 25459142 10.1016/j.mehy.2014.09.026

[aur1777-bib-0161] Tachibana, M. , Kagitani‐Shimono, K. , Mohri, I. , Yamamoto, T. , Sanefuji, W. , Nakamura, A. , … Taniike, M. (2013). Long‐term administration of intranasal oxytocin is a safe and promising therapy for early adolescent boys with autism spectrum disorders. Journal of Child and Adolescent Psychopharmacology, 23, 123–127. 23480321 10.1089/cap.2012.0048

[aur1777-bib-0162] Takagishi, H. , Takahashi, T. , Yamagishi, T. , Shinada, M. , Inukai, K. , Tanida, S. , … Kameda, T. (2010). Salivary testosterone levels and autism‐spectrum quotient in adults. Neuroendocrinology Letters, 31, 837. 21196912

[aur1777-bib-0163] Taurines, R. , Schwenck, C. , Lyttwin, B. , Schecklmann, M. , Jans, T. , Reefschläger, L. , … Romanos, M. (2014). Oxytocin plasma concentrations in children and adolescents with autism spectrum disorder: Correlation with autistic symptomatology. ADHD Attention Deficit and Hyperactivity Disorders, 6, 231–239. 24989441 10.1007/s12402-014-0145-y

[aur1777-bib-0164] Theodosis, D.T. , Koksma, J.J. , Trailin, A. , Langle, S.L. , Piet, R. , Lodder, J.C. , … Brussaard, A.B. (2006). Oxytocin and estrogen promote rapid formation of functional GABA synapses in the adult supraoptic nucleus. Molecular and Cellular Neuroscience, 31, 785–794. 16488155 10.1016/j.mcn.2006.01.006

[aur1777-bib-0165] Tierney, E. , Bukelis, I. , Thompson, R.E. , Ahmed, K. , Aneja, A. , Kratz, L. , & Kelley, R.I. (2006). Abnormalities of cholesterol metabolism in autism spectrum disorders. American Journal of Medical Genetics Part B: Neuropsychiatric Genetics, 141, 666–668. 10.1002/ajmg.b.30368 PMC2553243 16874769

[aur1777-bib-0166] Tierney, E. , Nwokoro, N.A. , & Kelley, R.I. (2000). Behavioral phenotype of RSH/Smith‐lemli‐opitz syndrome. Mental Retardation and Developmental Disabilities Research Reviews, 6, 131–134. 10899806 10.1002/1098-2779(2000)6:2<131::AID-MRDD7>3.0.CO;2-R

[aur1777-bib-0167] Tierney, E. , Nwokoro, N.A. , Porter, F.D. , Freund, L.S. , Ghuman, J.K. , & Kelley, R.I. (2001). Behavior phenotype in the RSH/Smith‐Lemli‐Opitz syndrome. American Journal of Medical Genetics, 98, 191–200. 11223857 10.1002/1096-8628(20010115)98:2<191::aid-ajmg1030>3.0.co;2-m

[aur1777-bib-0168] Tint, G.S. , Irons, M. , Elias, E.R. , Batta, A.K. , Frieden, R. , Chen, T.S. , & Salen, G. (1994). Defective cholesterol biosynthesis associated with the Smith‐Lemli‐Opitz syndrome. New England Journal of Medicine, 330, 107–113. 8259166 10.1056/NEJM199401133300205

[aur1777-bib-0169] Tordjman, S. , Anderson, G.M. , Kermarrec, S. , Bonnot, O. , Geoffray, M.M. , Brailly‐Tabard, S. , … Touitou, Y. (2014). Altered circadian patterns of salivary cortisol in low‐functioning children and adolescents with autism. Psychoneuroendocrinology, 50, 227–245. 25244637 10.1016/j.psyneuen.2014.08.010

[aur1777-bib-0170] Tordjman, S. , Anderson, G.M. , McBride, P.A. , Hertzig, M.E. , Snow, M.E. , Hall, L.M. , … Cohen, D.J. (1997). Plasma beta‐endorphin, adrenocorticotropin hormone, and cortisol in autism. Journal of Child Psychology and Psychiatry, 38, 705–715. 9315980 10.1111/j.1469-7610.1997.tb01697.x

[aur1777-bib-0172] Tordjman, S. , Ferrari, P. , Sulmont, V. , Duyme, M. , & Roubertoux, P. (1997). Androgenic activity in autism. American Journal of Psychiatry, 154, 1626–1627. 10.1176/ajp.154.11.1626-a 9356582

[aur1777-bib-0173] Travers, B.G. , Adluru, N. , Ennis, C. , Tromp, D.P. , Destiche, D. , Doran, S. , … Alexander, A.L. (2012). Diffusion tensor imaging in autism spectrum disorder: A review. Autism Research, 5, 289–313. 22786754 10.1002/aur.1243 PMC3474893

[aur1777-bib-0175] Ucuz, I.I. , Dursun, O.B. , Esin, I.S. , Özgeris, F.B. , Kurt, N. , Kiziltunç, A. , & Orbak, Z. (2014). The relationship between Vitamin D, autistic spectrum disorders, and cognitive development: Do glial cell line‐derived neurotrophic factor and nerve growth factor play a role in this relationship? International Journal of Developmental Disabilities, 61, 222–230.

[aur1777-bib-0176] Utur, C. , & Gurkan, C.K. (2014). Serum vitamin D and folate levels in children with autism spectrum disorders. Research in Autism Spectrum Disorders, 8, 1641–1647.

[aur1777-bib-0178] Van Rijn, S. , Stockmann, L. , Van Buggenhout, G. , van Ravenswaaij‐Arts, C. , & Swaab, H. (2014). Social cognition and underlying cognitive mechanisms in children with an extra X chromosome: A comparison with autism spectrum disorder. Genes, Brain and Behavior, 13, 459–467. 10.1111/gbb.12134 24655419

[aur1777-bib-0179] Waage‐Baudet, H. , Lauder, J.M. , Dehart, D.B. , Kluckman, K. , Hiller, S. , Tint, G.S. , & Sulik, K.K. (2003). Abnormal serotonergic development in a mouse model for the Smith–Lemli–Opitz syndrome: Implications for autism. International Journal of Developmental Neuroscience, 21, 451–459. 14659996 10.1016/j.ijdevneu.2003.09.002

[aur1777-bib-0180] Wassif, C.A. , Maslen, C. , Kachilele‐Linjewile, S. , Lin, D. , Linck, L.M. , Connor, W.E. , … Porter, F.D. (1998). Mutations in the human sterol delta7‐reductase gene at 11q12–13 cause Smith‐Lemli‐Opitz syndrome. American Journal of Human Genetics, 63, 55–62. 9634533 10.1086/301936 PMC1377256

[aur1777-bib-0181] Wassif, C.A. , Zhu, P. , Kratz, L. , Krakowiak, P.A. , Battaile, K.P. , Weight, F.F. , … Porter, F.D. (2001). Biochemical, phenotypic and neurophysiological characterization of a genetic mouse model of RSH/Smith–Lemli–Opitz syndrome. Human Molecular Genetics, 10, 555–564. 11230174 10.1093/hmg/10.6.555

[aur1777-bib-0182] Waterhouse, L. , Fein, D. , & Modahl, C. (1996). Neurofunctional mechanisms in autism. Psychological Review, 103, 457. 8759044 10.1037/0033-295x.103.3.457

[aur1777-bib-0183] Weisman, O. , Agerbo, E. , Carter, C.S. , Harris, J.C. , Uldbjerg, N. , Henriksen, T.B. , … Dalsgaard, S. (2015). Oxytocin‐augmented labor and risk for autism in males. Behavioural Brain Research, 284, 207–212. 25707712 10.1016/j.bbr.2015.02.028

[aur1777-bib-0184] Whitehouse, A.J. (2016). Commentary: Are we expecting too much from the extreme male brain theory of autism? A reflection on Kung et al. (2016). Journal of Child Psychology and Psychiatry, 57, 1463–1464. 27859346 10.1111/jcpp.12628

[aur1777-bib-0185] Whitehouse, A.J. , Holt, B.J. , Serralha, M. , Holt, P.G. , Kusel, M.M. , & Hart, P.H. (2012). Maternal serum vitamin D levels during pregnancy and offspring neurocognitive development. Pediatrics, 129, 485–493. 22331333 10.1542/peds.2011-2644

[aur1777-bib-0186] Whitehouse, A.J. , Mattes, E. , Maybery, M.T. , Dissanayake, C. , Sawyer, M. , Jones, R.M. , … Hickey, M. (2012). Perinatal testosterone exposure and autistic‐like traits in the general population: A longitudinal pregnancy‐cohort study. Journal of Neurodevelopmental Disorders, 4, 25. 23110806 10.1186/1866-1955-4-25 PMC3500651

[aur1777-bib-0200] Wing, L. (1981). Sex ratios in early childhood autism and related conditions. Psychiatry Research, 5, 129–137. 6945608 10.1016/0165-1781(81)90043-3

[aur1777-bib-0187] Wiznitzer, M. (2004). Autism and tuberous sclerosis. Journal of Child Neurology, 19, 675–679. 15563013 10.1177/08830738040190090701

[aur1777-bib-0188] Woods, A.G. , Wormwood, K.L. , Wetie, A.G.N. , Ryan, J.P. , & Darie, C.C. (2013). Proteomics and cholesterol in autism. Autism, 3, 2.

[aur1777-bib-0189] Wormwood, K.L. , Dupree, E.J. , Darie, C.C. , & Woods, A.G. (2014). Autism‐open access. Health Perspect, 120, 944–951.

[aur1777-bib-0190] Wust, S. , Wolf, J. , Hellhammer, D.H. , Federenko, I. , Schommer, N. , & Kirschbaum, C. (2000). The cortisol awakening response—Normal values and confounds. Noise Health, 2, 79–88. 12689474

[aur1777-bib-0191] Yang, C.J. , Tan, H.P. , Yang, F.Y. , Liu, C.L. , Sang, B. , Zhu, X.M. , & Du, Y.J. (2015). The roles of cortisol and pro‐inflammatory cytokines in assisting the diagnosis of autism spectrum disorder. Research in Autism Spectrum Disorders, 9, 174–181.

[aur1777-bib-0192] Zinke, K. , Fries, E. , Kliegel, M. , Kirschbaum, C. , & Dettenborn, L. (2010). Children with high‐functioning autism show a normal cortisol awakening response (CAR). Psychoneuroendocrinology, 35, 1578–1582. 20409644 10.1016/j.psyneuen.2010.03.009

